# Apathy scores in Parkinson’s disease relate to EEG components in an incentivized motor task

**DOI:** 10.1093/braincomms/fcae025

**Published:** 2024-02-09

**Authors:** Soojin Lee, Esther Song, Maria Zhu, Silke Appel-Cresswell, Martin J McKeown

**Affiliations:** Pacific Parkinson’s Research Centre, The University of British Columbia, Vancouver, BC V6T 2B5, Canada; Pacific Parkinson’s Research Centre, The University of British Columbia, Vancouver, BC V6T 2B5, Canada; Department of Psychiatry, The University of British Columbia, Vancouver, BC V6T 2A1, Canada; Pacific Parkinson’s Research Centre, The University of British Columbia, Vancouver, BC V6T 2B5, Canada; Department of Medicine, The University of British Columbia, Vancouver, BC V6T 1Z3, Canada; Pacific Parkinson’s Research Centre, The University of British Columbia, Vancouver, BC V6T 2B5, Canada; Division of Neurology, Department of Medicine, The University of British Columbia, Vancouver, BC V6T 2B5, Canada; Pacific Parkinson’s Research Centre, The University of British Columbia, Vancouver, BC V6T 2B5, Canada; Division of Neurology, Department of Medicine, The University of British Columbia, Vancouver, BC V6T 2B5, Canada

**Keywords:** Parkinson’s disease, apathy, event-related spectral perturbations, reward processing, beta desynchronization

## Abstract

Apathy is one of the most prevalent non-motor symptoms of Parkinson’s disease and is characterized by decreased goal-directed behaviour due to a lack of motivation and/or impaired emotional reactivity. Despite its high prevalence, the neurophysiological mechanisms underlying apathy in Parkinson’s disease, which may guide neuromodulation interventions, are poorly understood. Here, we investigated the neural oscillatory characteristics of apathy in Parkinson’s disease using EEG data recorded during an incentivized motor task. Thirteen Parkinson’s disease patients with apathy and 13 Parkinson’s disease patients without apathy as well as 12 healthy controls were instructed to squeeze a hand grip device to earn a monetary reward proportional to the grip force they used. Event-related spectral perturbations during the presentation of a reward cue and squeezing were analysed using multiset canonical correlation analysis to detect different orthogonal components of temporally consistent event-related spectral perturbations across trials and participants. The first component, predominantly located over parietal regions, demonstrated suppression of low-beta (12–20 Hz) power (i.e. beta desynchronization) during reward cue presentation that was significantly smaller in Parkinson’s disease patients with apathy compared with healthy controls. Unlike traditional event-related spectral perturbation analysis, the beta desynchronization in this component was significantly correlated with clinical apathy scores. Higher monetary rewards resulted in larger beta desynchronization in healthy controls but not Parkinson’s disease patients. The second component contained gamma and theta frequencies and demonstrated exaggerated theta (4–8 Hz) power in Parkinson’s disease patients with apathy during the reward cue and squeezing compared with healthy controls (HCs), and this was positively correlated with Montreal Cognitive Assessment scores. The third component, over central regions, demonstrated significantly different beta power across groups, with apathetic groups having the lowest beta power. Our results emphasize that altered low-beta and low-theta oscillations are critical for reward processing and motor planning in Parkinson’s disease patients with apathy and these may provide a target for non-invasive neuromodulation.

## Teaser

Lee *et al*. introduce a data-driven algorithm to analyse EEG data recorded during an incentivized motor task. Their findings reveal reduced low-beta (12–20 Hz) activity in Parkinson’s disease patients with apathy during reward processing and motor planning, irrespective of reward intensity, in contrast to those without apathy.

## Graphical Abstract

**Graphical Abstract:**
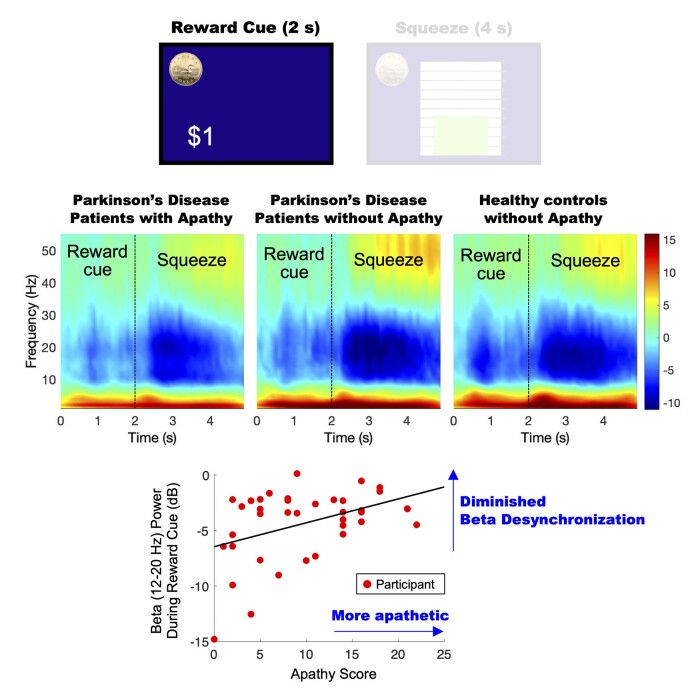

## Introduction

Apathy is one of the most debilitating non-motor symptoms of Parkinson’s disease (PD), manifesting as little or no goal-directed behaviour, along with multifaceted emotional and behavioural symptoms including reduced interest, lack of concern, emotional indifference and decreased initiation of activity.^1^ The ability to assess the value of potential rewards is a crucial aspect of motivation and goal-directed activity, and thus, the presence of apathy has a profoundly negative impact on people with PD, reducing their overall functioning and quality of life. While apathy in the non-PD population is mostly seen in the setting of depression, in PD apathy can be seen independently.^2^ PD-related apathy, which can affect between 17% and 70% of people,^1,3^ can result in withdrawal from physical activities, hobbies and social interactions, cognitive decline and poor engagement in rehabilitative treatments and impose a high burden on caregivers.

Currently, apathy is assessed by employing clinical assessment instruments such as the neuropsychiatric inventory, Lille apathy rating scale (LARS), apathy evaluation scale or apathy scale. While these apathy scales provide useful and reliable psychometric properties in PD,^4^ neuroimaging studies have provided additional insights into the anatomical and functional substrates implicated in apathy. One of the key characteristics of apathy involve alterations in the frontostriatal circuits, including the anterior cingulate cortex (ACC), ventral striatum (VS), nucleus accumbens and medial and lateral prefrontal cortex (PFC) and midbrain.^5-10^ For instance, PET studies have shown that apathy in PD is associated with reduced metabolic activity in the VS and medial frontal brain regions, including the ACC.^6,11,12^ PD patients with apathy also exhibit diminished dopamine receptor binding capacity in the bilateral VS^13^ and blunted dopamine release in the ACC, orbitofrontal cortex, dorsolateral PFC, thalamus and globus pallidus internal.^14^ In addition to the alterations in the frontostriatal circuits, several neuroimaging studies suggest that apathy in PD may stem from a severe dopamine depletion state in the mesocorticolimbic circuitry.^15^ Overall, these changes in neural systems have cascading effects on other brain regions, impacting the evaluation of costs and benefits (here, the ‘costs’ are assumed to reflect the effort associated with taking an action, and the ‘benefit’ involves the valuation of the expected potential rewards from the action^5,16,17^). For example, the activity in the ACC and its functional connectivity with the supplementary motor area have been found to predict behaviour apathy scores.^18^

**Figure 1:**
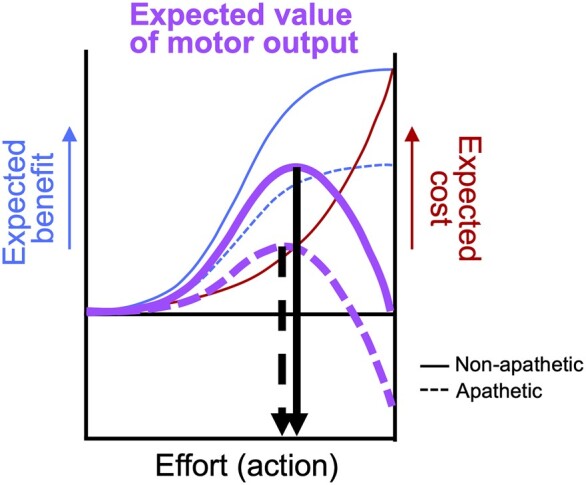
**A schematic diagram to illustrate allocation of behavioural effort determined by expected benefit and cost.** The diagram was adapted from the EVC theory^21^ (licenced under CC BY 4.0, http://creativecommons.org/licenses/by/4.0/). For a given reward (e.g. $1), people integrate information about the expected benefit (solid blue) and cost (solid red) to determine the expected value of motor output (solid purple), and then, they allocate the intensity of motor output (solid black arrow). For the same task and same amount of reward at stake, people with apathy may have diminished ‘drive’ by the potential benefit (dashed blue), which consequently alters the expected value of control (dashed purple) and intensity of effort (dashed black arrow). EVC, expected value of control.

Several studies have investigated abnormality in the cost–benefit valuation underlying motivational modulation of motor behaviour of PD patients with apathy. Heron *et al*.^19^ demonstrated that when participants were asked to make decisions on whether to accept or reject monetary rewards for exerting different levels of physical effort, PD patients with apathy made more rejections of offers (i.e. lower willingness to perform the physical effort) compared with both HCs and PD patients without apathy. This was predominantly observed in the trials with a low-level reward as the patients with apathy were found to be willing to exert effort to the same level as the patients without apathy if the reward was sufficiently high. This suggests that diminished ‘drive’ by the potential rewards for the action acts as a key factor in motivated behaviour in PD patients with apathy rather than an enhanced sensitivity to effort costs. Altered reward processing in PD patients with apathy was also observed in their blunting of pupillary dilation responses to incentives in saccadic eye movement tasks.^10,20^ Together, these studies suggest that alterations in processing incentivizing value of rewards play a crucial role in behavioural changes in PD patients with apathy (Fig. 1).

EEG and magnetoencephalography (MEG) studies offer valuable insights into the neuronal mechanisms underlying the reward processing and behaviours. One common approach involves analysing beta band oscillations (13–30 Hz) in motor and pre-motor areas.^22^ In healthy individuals, it has shown that the extent of beta suppression following reward cues is closely linked to motivational intensity^23^ and reflects the expected reward value.^24^ Despite a substantial body of evidence demonstrating abnormal beta oscillations in PD,^25-33^ their exact relationship with reward evaluation remains poorly understood. Notably, there is a scarcity of EEG studies investigating the neurophysiological mechanisms of apathy in PD, despite its high prevalence. To the best of our knowledge, only two EEG studies have examined reward processing in PD patients with apathy. One study^34^ measured time-locked event-related potentials (ERPs) to gain or loss feedback stimuli while the participants performed a modified Gehring’s gambling task. The study discovered significantly decreased amplitude of feedback-related negativity in PD patients with apathy compared with PD patients without apathy and HCs. The reduced amplitude was more notable for high-*win* than high-*loss* trials, suggesting a more selective impairment of neural processes under reward than punishment in PD patients with apathy. Another EEG study investigated alterations in spectral power and its relation to motivational motor behaviour in an incentivized motor task paradigm.^35^ Compared with PD patients without apathy and healthy individuals, PD patients with apathy were found to exhibit greater desynchronization in theta (4–7 Hz) and alpha (8–12 Hz) frequency bands during movement initiation and execution. The authors conjectured that it might reflect a compensatory phenomenon to counteract higher baseline theta and alpha power in the patients with apathy.

Here, we focused on apathy as a reduced motivation for goal-directed behaviour. We aimed to investigate characteristics of cortical oscillatory activity associated with incentive processing and assess whether there are changes associated with PD patients with apathy. Particularly, we introduce a novel data-driven method that can extract time-frequency domain features in multichannel EEG data that are highly relevant to the task and maximally correlated across participants. The proposed method has the advantage of (i) taking into account differences in spatial locations of event-related EEG dynamics across participants^36^ and (ii) obviating the necessity of confining power spectral analysis to specific frequency bands and EEG channels *a priori*. With this new data-driven method, we demonstrate that EEG dynamics related to processing reward cues are significantly attenuated in PD patients with apathy in the beta frequency band, which has long been linked to motivation and movement planning.^37-39^

## Materials and methods

### Participants

Patients diagnosed with idiopathic PD and age- and sex-matched HCs under the age of 85, with normal or corrected-to-normal vision, were recruited for this study. PD patients were recruited from the Movement Disorders Clinic at the University of British Columbia and prescribed with a stable dosage of an antiparkinsonian medication for at least 2 months before study enrolment. They were on their regular dopaminergic medication during the study visit. HCs were either spouses of patients or recruited from the community. The presence of depression and apathy was assessed using Beck’s depression inventory (BDI) and Starkstein apathy scale (SAS), respectively. The disease severity of the PD patients was assessed using the Movement Disorder Society-Unified Parkinson’s disease Rating Scale (UPDRS) Part III (motor examination). PD patients were classified into two groups, PDA+ (PD with apathy) and PDA− (PD without apathy), based on their SAS scores (see Demographics). All participants provided written informed consent before participation. The study protocol was approved by the Clinical Research Ethics Board at the University of British Columbia.

### Study protocol

Participants performed an incentivized motor task using a grip force transducer (Hand Dynamometer Logger Sensor NUL-237, NeuLog, USA) while seated comfortably in front of a 19 inch computer screen.

Before beginning the motor task, we determined each participant’s maximum voluntary contraction (MVC) to calibrate the target level of force used in the actual motor task to individuals. In the calibration process, participants were asked to squeeze the grip force transducer as hard as they could over three times using their dominant hand. The maximum of the three grips was recorded as the MVC. Participants were then asked to squeeze the grip force transducer to reach the red line on a graduated scale on the computer screen with real-time feedback, where the red line represented either 40%, 80% or 120% of the previously recorded MVC. The 120% grip force level was to ensure that the previously recorded MVC was the actual maximum participants could squeeze. For a total of nine trials, each target level of force was randomly presented three times. If participants squeezed harder than the recorded MVC in any of the three trials where the red line was 120% of the MVC, the maximum of the three 120% trials became the new recorded MVC. However, if participants, on any trial, reached or exceeded 120% of the first recorded MVC, then they were asked to restart the whole calibration process.

**Figure 2:**
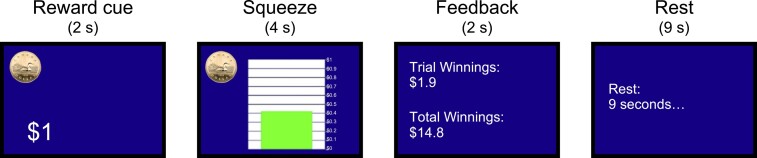
**Monetary incentive delay task. Reward cue: participants were presented with a certain amount of reward ($1, $10 or $50) for 2 s to indicate the maximum reward they could earn for the trial.** Squeeze: a graduated scale was displayed on the computer screen. The participants were instructed to squeeze a grip force transducer to increase the bar to the top and told that the amount of reward they would earn was proportional to how hard they squeezed within a 4 s window. Feedback: feedback on the reward they earned for the trial and total accumulated reward was given for 2 s. Rest: the participants were given 9 s to rest before the subsequent trial began.

The task consisted of showing participants a graduated scale on the computer screen and asking them to squeeze the grip force transducer for a monetary reward proportional to how hard they squeezed. Each trial consisted of the following steps (Fig. 2): first, participants were shown the maximum monetary reward they could earn for this trial for 2 s. The maximum monetary reward was one of $1, $10 or $50. Then, they were shown a graduated scale for 4 s and asked to squeeze the grip force transducer to earn the reward. The reward amount they could earn was proportional to how hard they squeezed while their grip force was shown on the graduated scale. At the top of the graduated scale was the maximum monetary reward shown at the start of the trial. To avoid ceiling effects, the graduated scale was set such that the 50% level was the participants’ MVC, as recorded in the calibration process. Then, participants were shown how much they had earned from the trial along with their current total winnings for 2 s. Finally, there was a 9 s rest before the subsequent trial. A total of 45 trials were performed, with the maximum monetary rewards of $1, $10 or $50 being presented 15 times each in randomized order.

### EEG recording and preprocessing

EEG data were recorded from 34 scalp electrodes using a 64-channel Quik-Cap (Neuroscan, VA, USA) and a Neuroscan SynAmps^2^ acquisition system (Neuroscan, VA, USA) at a sampling rate of 500 Hz. Recording electrodes were positioned according to the International 10–20 placement standard with one ground electrode (AFz) and one reference electrode located between Cz and CPz. Impedances were kept below 15 k Ω using Electro-Gel (Electrode-Cap International, OH, USA).

All EEG data were preprocessed offline using custom MATLAB scripts and functions from the open-source EEGLAB (https://sccn.ucsd.edu/eeglab) toolbox. Continuously recorded EEG signals were first bandpass filtered between 1 and 55 Hz using a two-way finite impulse response (FIR) filter (the ‘eegfilt’ function in EEGLAB) and then were re-referenced to average reference. Stereotypical artefacts, including ocular artefacts (EOG) and muscle tension, were removed using an automatic artefact rejection method^40^ based on independent component analysis (ICA).^41^ The EEG data were then segmented into non-overlapping 6 s epochs, each spanning 1 s of rest (i.e. baseline) before the beginning of the trial, 2 s of reward cue and 3 s of squeezing. The segmentation resulted in 45 epochs per participant, with 15 epochs per reward value ($1, $10 or $50).

### EEG time-frequency analysis

**Figure 3:**
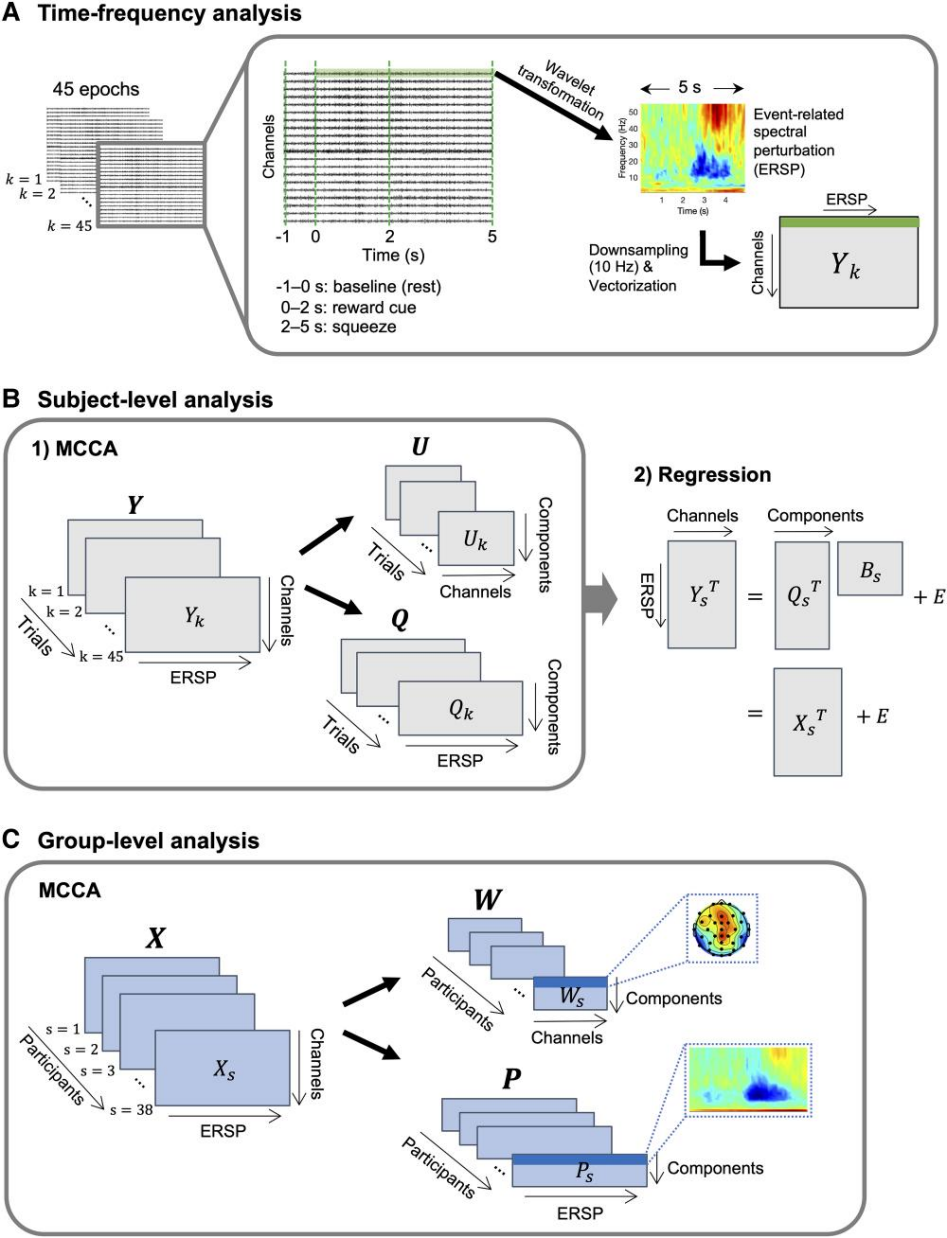
**EEG data analysis using wavelet transform and MCCA.** (**A**) A scalogram was obtained by applying a continuous wavelet transform to EEG timeseries. The ERSP scalogram was normalized based on the mean power during the baseline period, downsampled to 10 Hz and vectorized. (**B**) Subject-level MCCA was performed on the ERSP matrices ($Y_{k}\in R^{34\;\times \;2750}$) of 45 trials to extract 20 components ($Q_{k}\in R^{20\;\times \;2750}$) that are maximally correlated across the 45 trials. The original ERSP matrices ($Y_{k}$) and MCCA components ($Q_{k}$) were averaged, and a multiple linear regression was performed with each column in $Y_{s}^{T}$ as a dependent variable and the MCCA components $Q_{s}^{T}$ as independent variables. (**C**) Group-level MCCA was performed with the ERSP matrices ($X_{s}\in R^{34\;\times \;2750}$) derived from the subject-level analysis, resulting in three MCCA components. Each component consists of a common profile and corresponding weights on the EEG channels (depicted in dotted boxes). ERSP, event-related spectral perturbation; MCCA, multiset canonical correlation analysis.

After EEG segmentation, a continuous wavelet transform was applied to the 6 s EEG epochs using a seven-cycle, complex Morlet wavelet for a frequency range of 1–55 Hz (1 Hz steps). Spectral power for each frequency and timepoint was estimated by multiplying the complex signal from the wavelet transform by its complex conjugate. For each epoch, the mean baseline power ($P_{0}$) was computed by averaging the power during the baseline period (i.e. the first 1 s rest preceding a reward cue). Baseline-normalized event-related spectral perturbation (ERSP) was then computed by taking 10*log10 of the ratio of the spectral power to the mean baseline power. We then discarded the first 1 s and used the remaining 5 s task portion (i.e. 2 s reward cue + 3 s squeezing) of the ERSPs for further analysis (Fig. 3A).

### Multiset canonical correlation analysis

Multiset canonical correlation analysis (MCCA) is an extension of canonical correlation analysis (CCA) to allow for the joint analysis of more than two data sets. MCCA optimizes an objective function to achieve the maximum overall correlation of the canonical variates across the multiple data sets.^42,43^ In this study, we exploited MCCA to extract highly correlated time-frequency features in the baseline-normalized ERSPs across all trials and participants. Since the ERSPs represent task-related brain activities induced by the events (Fig. 2) that were identically timed across all trials and participants, we used MCCA to extract canonical variates of the ERSPs that are highly consistent across the trials and participants.

Figure 3 shows a schematic diagram explaining how MCCA was applied to the ERSP data. We first downsampled the ERSPs from the original sampling rate of 500–10 Hz (Fig. 3A). This resulted in an ERSP matrix with a dimension of 55 × 50 (frequency × time) per EEG channel and per epoch. Then, we vectorized the matrix and concatenated the ERSP vector for each channel along the row dimension, creating a 34 × 2750 ERSP matrix per epoch ($Y_{k}$; Fig. 3A).

Subject-level MCCA (Fig. 3B) was performed on the 45 ERSP matrices to extract the most consistent spectral patterns across all trials:


(1) U×Y=Q

Each MCCA component (i.e. canonical variate) consisted of a pair of weight (each row in $U_{k}\in R^{20\times 34}$; k=1,2,…,45 trials) and common profile (each row in $Q_{k}\in R^{20\times 2,750}$). The weight and common profiles are sorted in descending order according to the overall correlation across the 45 trials, such that the first row presents the MCCA component with the maximum overall correlation. The original ERSP data (Y) and common profile (Q) were then averaged across all trials, respectively.


(2) $$Y_{s}=\frac{1}{45}\sum_{k=1}^{45}Y_{k}$$

(3) $$Q_{s}=\frac{1}{45}\sum_{k=1}^{45}Q_{k}$$

A multiple linear regression was performed with each channel of $Y_{s}$ as the dependent variable and $Q_{s}$ as the independent variable. Finally, a mean ERSP matrix ($X_{s}$  $\in R^{34\times 2,750}$) was obtained for each subject *s* (s=1,2,…,38) by projecting the original mean scalogram ($Y_{s}$) onto the common profile ($Q_{s}$):


(4) $$X_{s}=(Q_{s}^{T}\times B_{s}{)}^{T}=B_{s}^{T}\times Q_{s}$$

The subject-level MCCA was performed based on the assumption that the components maximally correlated across different trials are more likely to be associated with task-relevant brain activities (‘signal’) than transient brain activities that appear in only one epoch or a small subset of the epochs or random noise. Therefore, we expected to obtain an ERSP matrix ($X_{s}$) with an improved signal-to-noise ratio by projecting the original mean scalogram ($Y_{s}$) onto the subspace of the MCCA components.

$X_{s}$ obtained from the subject-level MCCA was subsequently used as inputs to a group-level MCCA (Fig. 3C) to extract the time-frequency patterns that are maximally correlated across the participants:


(5) W×X=P

Each common profile in $P_{s}$ can be understood as a linear combination of the time-frequency features in $X_{s}$ weighted by the corresponding row in $W_{s}$ indicating the contribution of each channel. In this study, the most correlated time-frequency patterns across the participants were summarized into three components, which explained 90.1% variance of the input data X.

Each common profile from the group-level MCCA was averaged across the participants in each of the three groups (PDA+, PDA− and HC) for visualization purposes. The scalp maps were created by averaging the corresponding weights (W) across the participants.

### Conventional EEG spectral analysis

Additionally, we analysed the original ERSPs using a conventional method to compare the results with those obtained using the proposed MCCA approach. Conventionally, EEG electrodes of interest are pre-defined before any group-level analysis, often based on prior studies or domain knowledge about the brain regions involved in the neural processes of interest. We selected C3, CZ, C4, CP5, CP1, CPZ, CP2, CP6, P3, PZ and P4 electrodes as prior studies have indicated that spectral power in mu (8–12 Hz) and beta (12–30 Hz) frequency bands over the sensorimotor area are associated with motor processing.^44-47^

For each participant, we computed the grand mean ERSP by firstly taking the mean of the baseline-normalized ERSPs of 45 trials and then taking the mean over the selected electrodes. We also computed a mean ERSP over the 15 trials per incentive level to investigate whether the ERSP were modulated by the reward condition ($1, $10 or $50). The mean ERSPs were further used for statistical and correlation analyses.

### Statistical analysis

The group mean common profiles from MCCA were first visually inspected to determine the frequency bands and time periods that show clear differences between groups. The selected frequency bands and time periods were as follows:

Common profile 1:

- Low beta (12–20 Hz) during reward cue (0.3–1 s)
- Low beta during squeezing (2.5–4 s)

Common profile 2:

- Theta (4–8 Hz) during the entire epoch period (0–5 s)
- Gamma (30–55 Hz) during squeezing (2.5–5 s)

Common profile 3:

- Beta (12–30 Hz) during squeezing (2.5–4 s)

For each of the frequency bands and time periods of interest listed above, the mean spectral power was computed per participant. We performed a one-way ANOVA with group (PDA+, PDA−, HC) as a between-subject factor to test whether the mean power differs significantly across the groups. In addition, Tukey’s honestly significant difference (HSD) tests were conducted for *post hoc* comparisons.

To test whether the spectral power listed above varied with different reward levels, we computed the mean spectral power for each reward level per participant. In cases where the data were not normally distributed, we conducted a Friedman test to investigate differences in spectral power between different reward levels ($1/$10/$50) within each group. *Post hoc* comparisons were calculated using Wilcoxon signed-rank tests.

## Results

### Demographics

Twenty-seven PD patients and 13 HCs took part in the experiment. All participants were right-handed. The presence of apathy was determined by SAS scores ≥14. Accordingly, 13 out of the 27 PD patients were classified into PDA+ group and the remaining 14 PD patients were classified into PDA− group. All HCs had SAS scores below 14. One PDA− patient was excluded from the data analysis due to device malfunction, and one HC participant was excluded due to excessive artefacts in the EEG data. The demographic and clinical information of the remaining 13 PDA+, 13 PDA− and 12 HC participants are displayed in Table 1.

**Table 1:** Demographic comparison of the participants

	HC	PD	*P*-value (HC versus PD)	PDA−	PDA+	*P*-value (PDA− versus PDA+)
*N*	12	26	n/a	13	13	n/a
Age (years)	70.4 (±7.3)	68.6 (±4.9)	0.377	67.5 (±5.1)	69.8 (±4.6)	0.240
Sex (M/F)	5/7	14/12	0.485	6/7	8/5	0.253
SAS	5.7 (±4.7)	11.8 (±6.0)	0.004*	6.6 (±3.0)	16.9 (±2.9)	4.7 × 10^−9^*
LARS	−31.3 (±2.5)	−25.0 (±6.6)	0.004*	−28.8 (±5.2)	−21.3 (±5.9)	0.002*
BDI	4.7 (±4.4)	8.8 (±6.2)	0.043*	4.8 (± 4.0)	12.8 (± 5.4)	2.4 × 10^−4^*
MoCA	26.1 (±2.2)	26.4 (±2.2)	0.662	26.5 (±1.8)	26.4 (± 2.6)	0.931
MDS-UPDRS III	n/a	26.7 (±7.9)	n/a	26.0 (±8.3)	27.3 (±7.8)	0.682
Hoehn and Yahr	n/a	2.3 (±0.5)	n/a	2.1 (±0.4)	2.5 (±0.6)	0.065
Levodopa equivalence (mg)	n/a	1141.5 (±739.2)	n/a	1235.8 (±872.7)	1047.1 (±598.0)	0.526
Antidepressant use [*N* (%)]	n/a	13 (50%)	n/a	7 (53.9%)	6 (46.2%)	0.695
Cholinesterase inhibitor use [*N* (%)]	n/a	1	n/a	0 (0%)	1 (7.7%)	0.308

Numbers in brackets represent standard deviations. All participants are right-handed. SAS, Starkstein apathy scale; LARS, Lille apathy rating scale; BDI, Beck’s depression inventory; MoCA, Montreal Cognitive Assessment; MDS-UPDRS, Movement Disorder Society-Unified Parkinson’s Disease Rating Scale; n/a, not applicable. *indicates significant a *P*-value.

### Subject-level analysis

**Figure 4:**
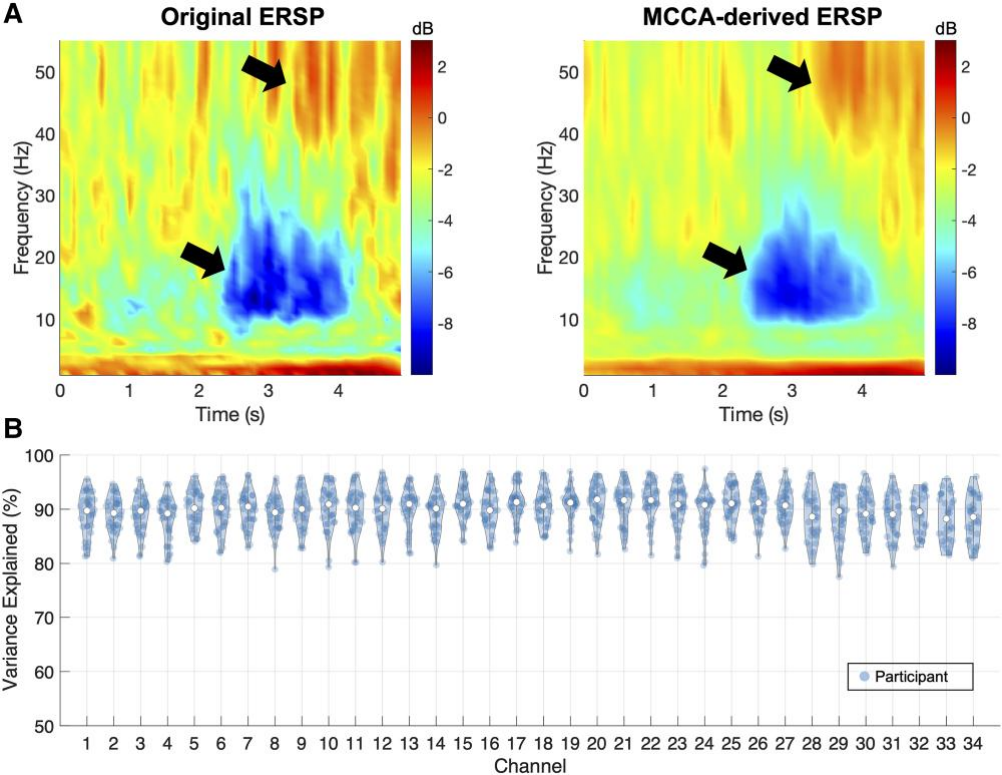
**Qualitative and quantitative comparison of the original ERSP and ERSP derived from MCCA.** (**A**) The trial mean ERSP of channel FP1 from a representative participant. The MCCA-derived ERSP (i.e. $X_{s}$ in Fig. 3) preserves task-relevant spectral changes in the original ERSP ($Y_{s}$) as denoted by arrows. (**B**) The distribution of the variance of the original ERSP explained by the MCCA-derived ERSP computed for each participant per channel. ERSP, event-related spectral perturbation; MCCA, multiset canonical correlation analysis.

To investigate the effects MCCA has on the ERSP data, we performed qualitative and quantitative comparisons between the mean ERSP obtained from the original data ($Y_{s}$) and MCCA ($X_{s}$). Figure 4A shows the qualitative comparison of the mean ERSP of channel FP1 obtained from a representative participant. It can be seen that the MCCA-derived ERSP (right panel in Fig. 4A) preserves the task-relevant brain activities, demonstrating the beta desynchronization and subsequent gamma synchronization occurred during squeezing (2–5 s). The other spectral changes appeared to be relatively indistinct, making the MCCA-derived ERSP less noisy compared with the original ERSP. Quantitative comparisons are shown in Fig. 4B where the percentage of the variance of the original ERSP explained by the MCCA-derived ERSP is shown. We found that the MCCA-derived ERSP preserved in average 89.8 ± 3.7% (mean ± standard deviation) of the variance contained in the original ERSPs.

### Group-level analysis

We investigated whether the ERSP obtained from the group-level MCCA ($P_{s}$; s= 1, 2, …, 38) had stronger between-subject correlations compared with the original ERSP ($Y_{s}$; s= 1, 2, …, 38). The between-subject correlations were computed as the correlations of ERSP between every pair of two participants for each channel (Supplementary Fig. 1A) or component (Supplementary Fig. 1B). It was found that the between-subject correlations of the original ERSP ranged from r= 0.35 ± 0.16 to r= 0.45 ± 0.15 across channels. The grand mean of the between-subject correlations of all channels was r= 0.40 ± 0.18. On the other hand, the between-subject correlations of the first MCCA ERSP were found to be *r* = 0.74 ± 0.08 (Supplementary Fig. 1B), which was significantly higher than those of the original ERSP (*D* = 0.83, *P* < 0.001; two-sample Kolmogorov–Smirnov test). The second MCCA ERSP had the between-subject correlations of r= 0.54 ± 0.16, which was also significantly higher than those of the original ERSP (*D* = 0.32, *P* < 0.001; two-sample Kolmogorov–Smirnov test). In contrast, the between-subject correlations for the third MCCA ERSP were significantly lower than those of the original ERSP (*D* = 0.39, *P* < 0.001; two-sample Kolmogorov–Smirnov test).

### First group-level MCCA component

**Figure 5:**
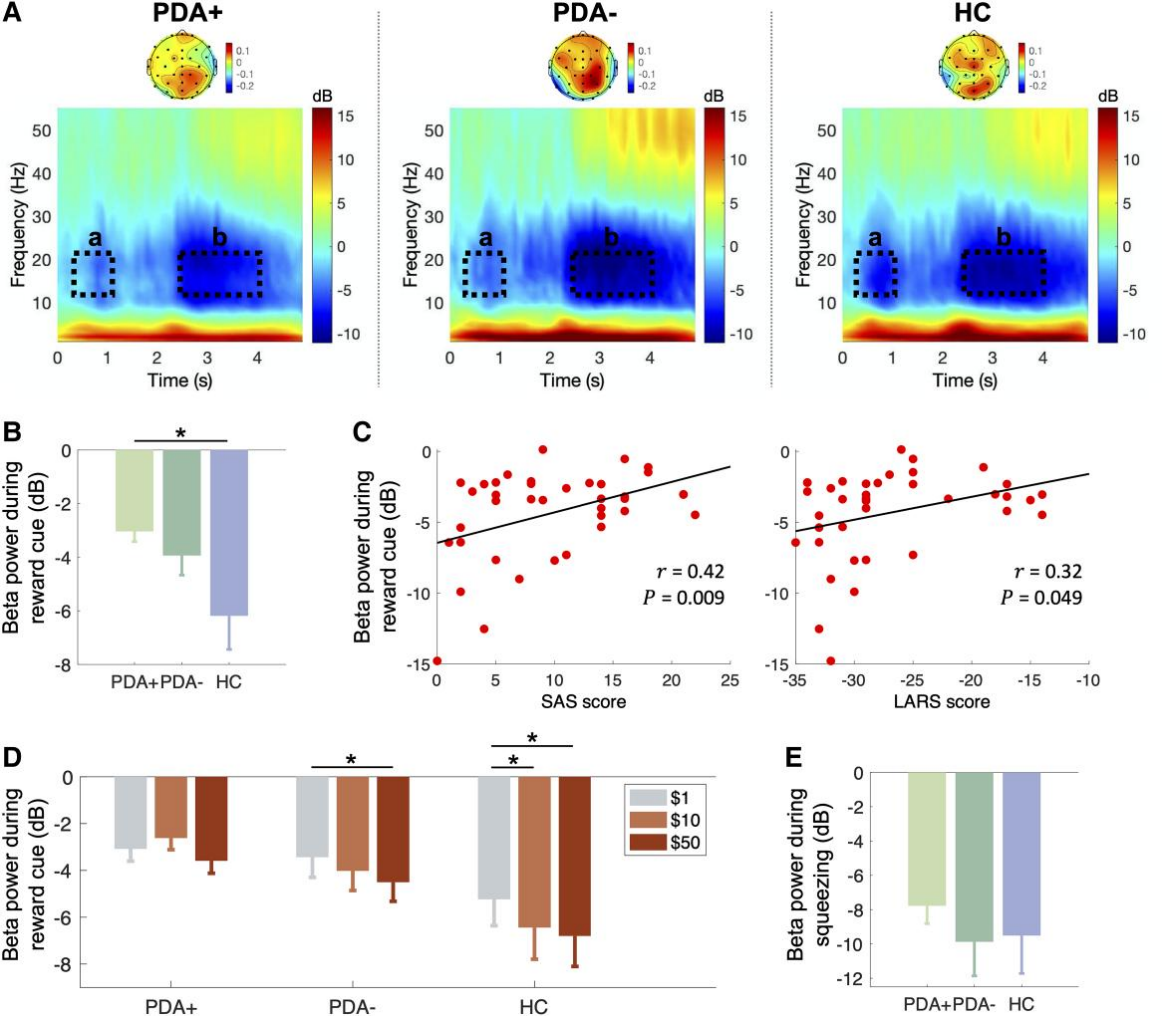
**First group-level MCCA component.** (**A**) The ERSP and its weights across EEG channels are demonstrated as a scalogram (bottom) and scalp map (top) for each group. The low-beta (12–20 Hz) frequency band during a reward cue (a: 0.3–1 s) and squeezing (b: 2.5–4 s) is denoted as dotted boxes. (**B**) Group comparison of the beta power during a reward cue (statistics: one-way ANOVA, Tukey’s honestly significant difference test). (**C**) Correlations between the beta power during a reward cue and clinical apathy scores (SAS and LARS) of all the participants. (**D**) The beta power during the reward cue is presented per reward level for each group (statistics: Friedman test, Wilcoxon signed-rank test). (**E**) Group comparison of the beta power during squeezing (statistics: one-way ANOVA). *P < 0.05. ERSP, event-related spectral perturbation; HC, healthy controls (*N* = 12); LARS, Lille apathy rating scale; MCCA, multiset canonical correlation analysis; PDA+, Parkinson’s disease patients with apathy (*N* = 13); PDA−, Parkinson’s disease patients without apathy (*N* = 13); SAS, Starkstein apathy scale.

Figure 5A shows the ERSP (P) at the bottom and its weights across EEG channels (W) at the top for the first group-level MCCA component. The results are demonstrated as the group means of the participants in the PDA+, PDA− and HC groups, respectively. For all three groups, the EEG channels in the parietal region contributed most strongly to the ERSP, followed by the EEG channels in the frontal region. The spectral power changes in the ERSP were characterized by the increased power in the delta (<4 Hz, 0–5 s) and gamma (30–55 Hz, >3 s) bands and the decreased power in the beta band (12–30 Hz, >0.3 s). The beta power changes, particularly in the low-beta frequency band (12–20 Hz), were of particular interest because (i) the temporal profile corresponded well to the timing of task stimuli (i.e. greater attenuation immediately after the reward cue presented at *t* = 0 s and target force level presented at *t* = 2 s) and (ii) prior studies have indicated that low-beta oscillations are implicated in impaired movement initiation in PD.^48,49^ Therefore, we investigated changes in the low-beta band power (Fig. 5A) during a reward cue (a: 0.3–1 s) and squeezing (b: 2.5–4 s) to find out whether there is a significant difference across the PDA+, PDA− and HC groups.

A one-way ANOVA analysis demonstrated a significant group difference in the beta desynchronization during reward cue presentation [*F*(2,35) = 3.64, *P* < 0.05]. In addition, Tukey’s HSD *post hoc* multiple comparison tests revealed a greater beta desynchronization in the HC group compared with the PDA+ group (*P*_HSD_ < 0.05; Fig. 5B), whereas there was no significant difference between PDA+ and PDA− groups (*P*_HSD_ = 0.72) and PDA− and HC groups (*P*_HSD_ = 0.16).

We further evaluated the clinical significance of the diminished beta desynchronization observed in the apathy group using the SAS and LARS apathy scores (Fig. 5C). The beta desynchronization during the reward cue was positively correlated with SAS (*r* = 0.42, *P* < 0.01; *N* = 38) and LARS (*r* = 0.32, *P* < 0.05; *N* = 38) scores, respectively, indicating that the more apathetic the participants are, the less beta desynchronization they exhibit during the reward cue. We further investigated whether the beta desynchronization level was modulated by the values of the monetary incentive (i.e. $1, $10 or $50). Friedman tests showed a significant effect of the monetary value on the beta desynchronization for the PDA− ($\chi_{\left(2\right)}^{2}$ = 6.4, *P* < 0.05) and HC ($\chi_{\left(2\right)}^{2}$ = 6.9, *P* < 0.05) groups, but not for the PDA+ group ($\chi_{\left(2\right)}^{2}$ = 2.0, *P* = 0.37) (Figure 5D). *Post hoc* Wilcoxon signed-rank tests revealed that $50 reward resulted in a greater amount of beta desynchronization in both PDA− (*Z* = 2.34, *P* < 0.05) and HC (*Z* = 2.35, *P* < 0.05) participants. In contrast, $10 reward resulted in greater beta desynchronization in the HC participants only (*Z* = 2.27, *P* < 0.05).

The beta desynchronization during squeezing (2.5–4 s) did not significantly differ across the three groups [one-way ANOVA: *F*(2,35) = 0.4, *P* < 0.05; Fig. 5E] nor correlated with the participants’ apathy scores (SAS: *r* = 0.20; *P* = 0.24; *N* = 38; LARS: *r* = 0.04; *P* = 0.83; *N* = 38).

### Second group-level MCCA component

**Figure 6:**
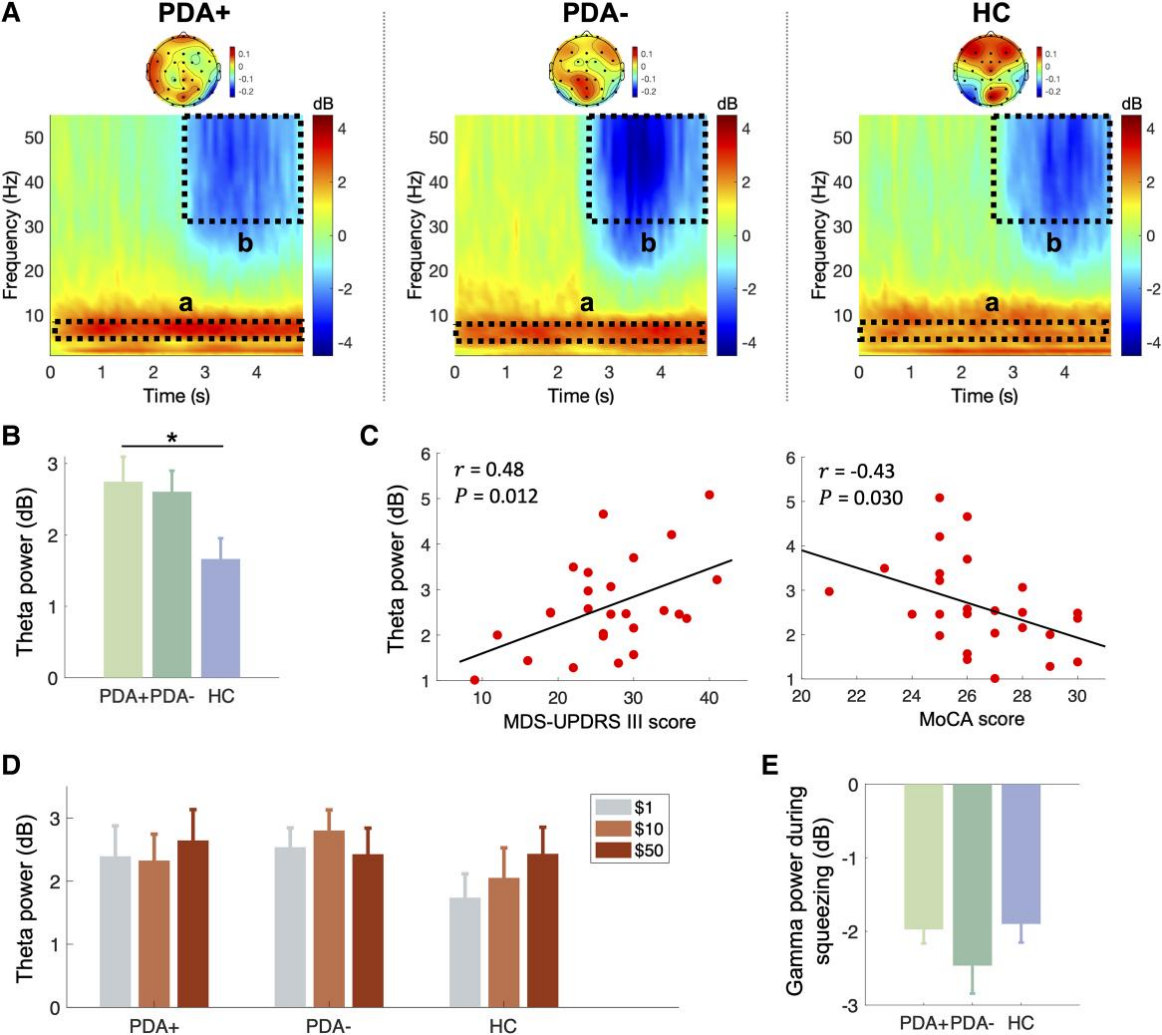
**Second group-level MCCA component.** (**A**) The ERSP and its weights across EEG channels are demonstrated as a scalogram (bottom) and scalp map (top) for each group. The theta (4–8 Hz) frequency band during the reward cue and squeezing (a: 0–5 s) and gamma (30–55 Hz) frequency band during squeezing (b: 2.5–5 s) are denoted as dotted boxes. (**B**) Group comparison of the theta power during the reward cue and squeezing (statistics: one-way ANOVA, Tukey’s honestly significant difference test). (**C**) Correlation between the theta power and MDS-UPDRS Part III scores (left) and between the theta power and MoCA scores (right) of the PD participants. (**D**) The theta power is presented per reward level for each group (statistics: Friedman test). (**E**) Group comparison of the gamma power during squeezing (statistics: one-way ANOVA). *P < 0.05. ERSP, event-related spectral perturbation; HC, healthy controls (*N* = 12); LARS, Lille apathy rating scale; MCCA, multiset canonical correlation analysis; MDS-UPDRS, Movement Disorder Society-Unified Parkinson’s Disease Rating Scale; MoCA, Montreal Cognitive Assessment; PD, Parkinson’s disease; PDA+, Parkinson’s disease patients with apathy (*N* = 13); PDA−, Parkinson’s disease patients without apathy (*N* = 13); SAS, Starkstein apathy scale.

The ERSP and weights of the second MCCA component are shown in Fig. 6A. The weights presented in the scalp maps showed the spectral changes in the ERSP were associated with frontal and parietal brain regions. The ERSP was featured with the increased theta (4–8 Hz) power over the entire period of the reward cue and squeezing and attenuated gamma (30–55 Hz) power during squeezing (2.5–5 s) compared with the baseline. A one-way ANOVA analysis showed a significant group difference in the theta power [*F*(2,35) = 3.4, *P* = 0.045; Fig. 6B], with a significantly higher theta power found in the PDA+ group compared with the HC group (*P*_HSD_ = 0.048). The PDA− group tended to have a higher theta power than the HC group, but this difference did not reach statistical significance (*P*_HSD_ = 0.10). The increased theta power observed in the PD participants in both the PDA+ and PDA− groups was found to be significantly correlated with their Movement Disorder Society-Unified Parkinson’s Disease Rating Scale (MDS-UPDRS) III scores (*r* = 0.48, *P* = 0.012, *N* = 26), indicating that the patients with higher disease severity exhibited exaggerated theta power during the reward cue and squeezing. We also found that the theta power was negatively correlated with the Montreal Cognitive Assessment (MoCA) scores of the PD participants (*r* = −0.43, *P* = 0.030, *N* = 26), suggesting that the excessive theta power may be also related to cognitive decline in PD. When all participants were taken into account, the correlation between the theta power and MoCA scores became no longer statistically significant (*r* = 0.26, *P* = 0.116, *N* = 38).

For the gamma power during squeezing, we did not find a significant difference across the three groups [one-way ANOVA: *F*(2,35) = 1.16, *P* = 0.330; Fig. 6E].

### Third group-level MCCA component

The ERSP of the third MCCA component was found to be associated with the EEG electrodes located in the central region (Supplementary Fig. 2A). The ERSP showed enhanced gamma (30–55 Hz) power during reward cue (0–2 s), followed by suppression of beta (12–30 Hz) power during squeezing (2.5–4 s). A one-way ANOVA revealed no significant difference in gamma power [*F*(2,35) = 3.4, *P* < 0.05] across the three groups. In contrast, the beta power was found to be significantly different across the groups [one-way ANOVA: *F*(2,35) = 3.68, *P* < 0.05], with PDA+ group having a significantly lower beta power compared with the HC group (*P*_HSD_ < 0.05) during squeezing.

### Grip force response

**Figure 7:**
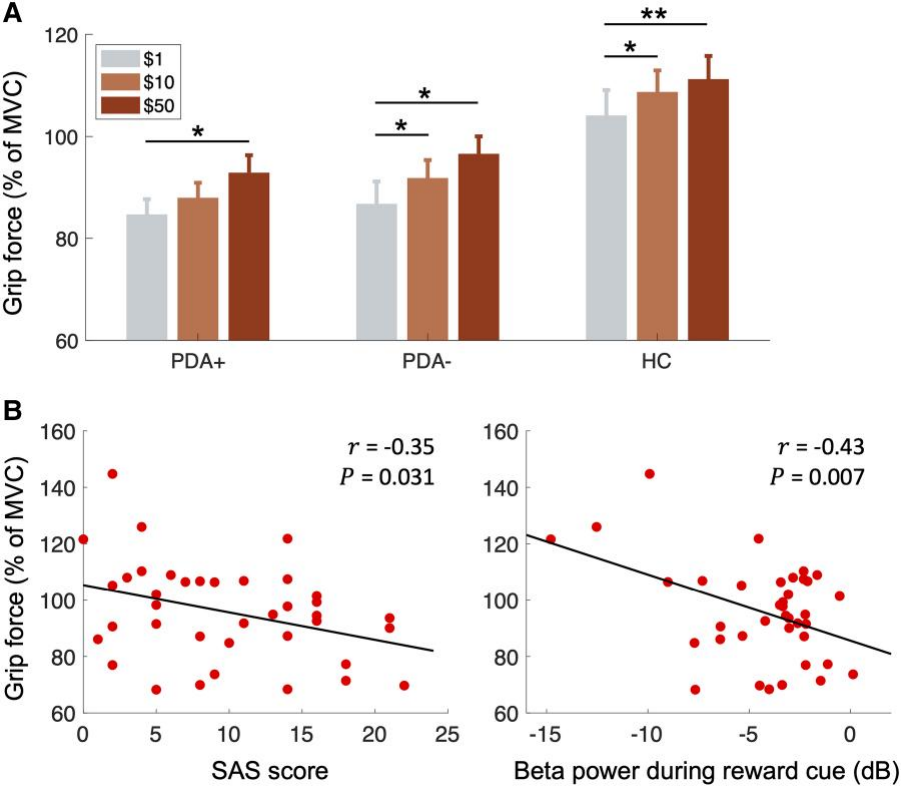
**Grip force response results.** The grip force is expressed as a percentage of the total MVC. (**A**) Group comparisons of the grip force responses to reward cue ($1, $10 or $50) (statistics: repeated-measure ANOVA, Tukey’s honestly significant difference test). (**B**) Left: Correlation between the participants’ mean grip force responses and SAS scores. Right: Correlation between the mean grip force responses and low-beta (12–21 Hz) power during reward cue (denoted as panel **A** in Fig. 5A). *P < 0.05; **P < 0.01. HC, healthy controls (*N* = 12); MVC, maximum voluntary contraction; PDA+, Parkinson’s disease patients with apathy (*N* = 13); PDA−, Parkinson’s disease patients without apathy (*N* = 13); SAS, Starkstein apathy scale.

The behaviour results of the grip force responses to the reward cue were analysed using a two-way mixed ANOVA with reward ($1/$10/$50) as the within-subject factor and group (PDA+/PDA−/HC) as the between-subject factor. The results showed significant main effects of reward [*F*(2,70) = 19.2, *P* < 0.0001] and group [*F*(2,35) = 5.62, *P* < 0.01], but not the interaction between reward and group [*F*(4,70) = 34.9, *P* = 0.889]. We further explored how the grip force behaviour varied across different reward levels by conducting a repeated-measure ANOVA with reward [$1/$10/$50] as the within-subject factor for each group. The results revealed that grip force significantly varied in response to varying reward levels for all groups [PDA+: *F*(2,24) = 5.49, *P* < 0.05; PDA−: *F*(2,24) = 7.77, *P* < 0.01; HC: *F*(2,22) = 9.41, *P* < 0.01; Fig. 7A]. Tukey’s HSD *post hoc* tests showed that the $50 reward induced greater grip force compared with the $1 reward in all three groups (PDA+: *P*_HSD_ < 0.05; PDA−: *P*_HSD_ < 0.05; HC: *P*_HSD_ < 0.01). The $10 reward led to increased grip force in the PDA− (*P*_HSD_ < 0.05) and HC (*P*_HSD_ < 0.05) groups, but this effect was not observed in the PDA+ group (*P*_HSD_ = 0.19).

We investigated whether participants’ average grip force during the experiment was associated with their apathy scores and the ERSP obtained from the group-level MCCA. Specifically, we used the low-beta ERSP of the first MCCA component during reward cue (Fig. 5). The mean grip force (across all trials) of the participants was found to be negatively correlated with both the SAS scores (*r* = −0.35, *P* < 0.05, *N* = 38; Fig. 7B) and low-beta power (*r* = −0.43, *P* < 0.01, *N* = 38; Fig. 7B) during reward cue, indicating that the participants with greater apathy severity used lower grip force and had higher low-beta power (i.e. smaller amount of beta desynchronization) during the reward cue.

### ERSP results from conventional analysis

Supplementary Figure 3A shows the group mean ERSPs over the central–parietal channels (C3, CZ, C4, CP5, CP1, CPZ, CP2, CP6, P3, PZ and P4). The low-beta power was found to be suppressed during reward cue (a: 0.3–1 s) and squeezing (b: 2.5–4 s), similar to the results found in the first group-level MCCA component (Fig. 5A). One-way group ANOVA revealed a significant group difference in the beta power during reward cue [*F*(2,35) = 5.23, *P* < 0.05; Supplementary Fig. 3B]. The amount of beta desynchronization in the HC group was greater than the PDA− group (*P*_HSD_ < 0.01) but not the PDA+ group (*P*_HSD_ = 0.080). No significant correlation was found between the beta power during reward cue and apathy scores (SAS: *r* = 0.04, *P* = 0.808; LARS: *r* = 0.06, *P* = 0.724; Supplementary Fig. 3C). We did not find a significant correlation of the beta power with the apathy scores even when we computed the beta power over every possible subset of the central–parietal channels as shown in Supplementary Fig. 3D (SAS: *r* = 0.04 ± 0.05; LARS: *r* = 0.06 ± 0.04), which was in contrast to the significant correlations found with the first MCCA component (Fig. 5C). Furthermore, it was found that the beta power did not vary according to different reward cues (Supplementary Fig. 3E).

No significant group difference was found for the beta power during squeezing [one-way ANOVA: *F*(2,35) = 0.98, *P* = 0.386; Supplementary Fig. 3F].

### Effects of sampling rate on MCCA

The MCCA results discussed in previous sections were based on ERSPs downsampled to 10 Hz, a step taken to reduce computational burden and data storage space. To assess the consistency of our findings at a higher sampling rate, we downsampled the ERSPs from the original 500 to 50 Hz and applied the same subject-level and group-level MCCA procedures outlined in Fig. 3 to the 50 Hz ERSP data.

The subject-level analysis results (Supplementary Fig. 4A) closely mirrored those obtained from ERSPs sampled at 10 Hz, affirming that the MCCA-derived ERSP preserves the task-relevant cortical oscillations. The MCCA-derived ERSPs explained 90.5 ± 3.4% of the variance in the original ERSPs (Supplementary Fig. 4B). The grand mean of between-subject correlations for the 50-Hz ERSPs across all channels (Supplementary Fig. 5A) was r= 0.40 ± 0.18, while the between-subject correlations for the first, second and third MCCA-derived ERSPs (Supplementary Fig. 5B) were r= 0.75 ± 0.09, 0.54 ± 0.17 and 0.17 ± 0.25, respectively. These results closely paralleled the results shown in Supplementary Fig. 1B. Supplementary Figures 6–8 show the group-level MCCA components derived from the 50 Hz ERSPs, affirming the consistency of the group-level results in comparison with the MCCA results obtained from 10 Hz ESRPs, as previously presented in Figs. 5 and 6 and Supplementary Fig. 2.

## Discussion

This study investigated the brain oscillations recorded in EEG to study the neurophysiological processes underlying motivational control of goal-directed movements and how it is associated with the clinical phenotype of apathy in PD. We investigated ERSPs induced by the reward cues and squeezing actions using a novel data-driven method utilizing MCCA to extract spectral features that are maximally correlated across trials and participants and therefore provided more robust representations of the brain dynamics related to the monetary incentive task.

An important finding of the present study was the ability of the proposed method to disentangle different spectral perturbations into separate components. The first MCCA component demonstrated strong modulation of beta oscillations during the performance of the incentivized task. The low-beta (12–20 Hz) power attenuated during reward cues, and the amount of suppression was inversely correlated with clinical apathy scores. That is, the participants with greater apathy severity showed impaired beta suppression to reward cues. Furthermore, the beta suppression was sensitive to the valence of potential reward in the HC group, but this was observed between $1 and $50 cues in the PDA− group and not observed at all in the PDA+ group. In contrast, the amount of beta suppression computed using a conventional method using the same frequency range and time segment was not found to be correlated with the apathy scores nor was it sensitive to the level of potential reward, even in HCs. These results suggest that the proposed MCCA approach could be a valuable way to derive ERSPs that are more robust to noise and task-irrelevant neural processing and reflect the essential neural processes underlying the task.

Our result of the beta power mediated by motivational salience, as revealed by the first MCCA component, is corroborated by a study^50^ that investigated the effects of positive and negative monetary incentives on beta oscillations during an incentivized goal-directed reaching task. The amount of beta suppression during the reward cue was found to be significantly greater in reward trials compared with neutral and punish trials. It also scaled with the rewards at stake and significantly correlated with the participants’ movement time. These results support the notion that pre-movement beta suppression reflects the neural processes underlying incentive-driven motor planning, which is disrupted in PD patients with apathy.

Beta oscillations have been predominantly implicated in sensorimotor processing, evidenced by a large number of prior studies reporting that the beta oscillations desynchronize during motor planning prior to movement onset^37,45,51-54^ and during movement execution.^54-56^ The sensorimotor cortex and basal ganglia are considered the principal sources for the emergence of beta desynchronization.^39^ Extensive studies have tried to elucidate the functional roles of beta oscillations in the sensorimotor system. One popular hypothesis is that beta oscillations relate to mechanisms that maintain the ‘status-quo/idling status’ of the sensorimotor system, compromising neuronal processing of new movements.^57-61^ In this context, beta activity needs to be inhibited to allow the initiation of motor planning and execution.^39^ A large body of evidence has been in support of this hypothesis, demonstrating that people with movement disorders like PD that impair motor initiation exhibit excessive power and coherence in the ‘antikinetic’ beta bands during the resting state^26-28,62^ and diminished amount of beta suppression during motor planning and execution.^29,63-65^ In this context, our result demonstrating reduced beta desynchronization during reward cues in PD participants may partially reflect the pathological changes in the neural processing necessary for motor planning for an intended movement.

In the PD participants with apathy, we observed an overall reduction in the amount of beta suppression (Fig. 5B), and their beta suppression did not scale with the reward level compared with the PD participants without apathy and HCs (Fig. 5D). Behaviourally, the PD participants with apathy also displayed reduced sensitivity to changes in reward level (Fig. 7A). Our results are aligned with the findings from prior research that PD patients with apathy are less likely to engage in physical effort for a reward, especially when the reward is relatively small, as compared with those PD patients without apathy.^19,20^ A recent study^10^ has investigated whether this diminished reward sensitivity is present in PD patients with apathy regardless of motor preparation. The authors found that it only manifests when actions are required to achieve rewarding goals. This suggests that the interaction between reward evaluation and the initiation of goal-directed action plays a crucial role in apathy in PD. Unfortunately, our study design does not allow us to discern whether our findings reflect impairments in sensitivity to reward (i.e. reward evaluation) or translating reward evaluation into effort (i.e. motor planning) or a combination of both. In light of the insights from the aforementioned studies, the aberrant beta suppressions we observed in PD patients with apathy may reflect pathological neural mechanisms underlying both reward evaluation and motor planning.^10^ To the best of our knowledge, no study to date has explicitly investigated the role of cortical beta oscillations in PD patients with apathy. Therefore, further research is warranted in the future to delve deeper into and substantiate this hypothesis.

In the second MCCA component, we observed that frontal–parietal theta power was generally higher in PD participants than HCs throughout the reward cue and squeezing periods. In contrast to the beta power of the first MCCA component, the theta power did not correlate with the incentive level or grip force responses. Based on prior studies, we conjecture that the elevated theta power we observed may be related to a general slowing of background cortical oscillatory activity in PD patients. As reported in a recent systematic review examining 19 studies with 312 PD patients and 277 controls,^66^ a large body of EEG studies on PD have demonstrated slowing of cortical activity, which is reflected as an increase of spectral power of delta and theta oscillations or a slower peak frequency. The EEG slowing in PD appears to be particularly associated with cognitive impairment^67^ as PD patients with mild cognitive impairment (MCI) or dementia have been reported to have more pronounced EEG slowing compared with PD patients with normal cognitive function^68^ or even Alzheimer’s disease patients with a similar degree of overt dementia.^69-71^ In support of this notion, we found a significant correlation between the elevated theta power and lower MoCA scores in the PD participants (Fig. 6C).

The other prominent feature of the second MCCA component was the suppression of gamma oscillations. Gamma rhythms (30–50 Hz) have been implicated in various cognitive tasks such as working memory, mental arithmetic, visuomotor coordination and selective attention.^72,73^ Prior studies on PD patients with dementia or MCI often report EEG slowing in the patients accompanied by decreased power of gamma oscillations,^74-76^ and similarly, we found that the theta power was inversely correlated with the gamma power (*r* = −0.40, *P* = 0.013). Since the gamma suppression was extracted together with the elevated theta power in the same MCCA component, we surmise it may also reflect some cognitive aspects of brain activity.

To conclude, the present study provides a critical link between the clinical features of apathy in PD and altered oscillatory dynamics, including beta, gamma and theta band frequencies. Given that neuromodulation can augment desynchronization in certain circumstances,^77^ our results may allow for specific non-pharmacological targeting of apathy symptoms in PD.

## Supplementary Material

fcae025_Supplementary_Data

## Data Availability

The data and custom written analysis code that support the findings of this study are available on request from the corresponding author.

## References

[1] Pluck GC, Brown RG. Apathy in Parkinson’s disease. J Neurol Neurosurg Psychiatry. 2002;73(6):636–642. 12438462 10.1136/jnnp.73.6.636 PMC1757348

[2] Kirsch-Darrow L, Marsiske M, Okun MS, Bauer R, Bowers D. Apathy and depression: Separate factors in Parkinson’s disease. J Int Neuropsychol Soc. 2011;17(6):1058–1066. 22040900 10.1017/S1355617711001068 PMC3302577

[3] Muhammed K, Manohar S, Husain M. Mechanisms underlying apathy in Parkinson’s disease. Lancet. 2015;385(Suppl 1):S71. 26312893 10.1016/S0140-6736(15)60386-5

[4] Lee B, Gleason C, Umucu E. Clinical utility and psychometric properties of the apathy evaluation scale. Rehabil Psychol. 2020;65(3):311–312. 32804534 10.1037/rep0000356 PMC8127218

[5] Le Heron C, Apps MAJ, Husain M. The anatomy of apathy: A neurocognitive framework for amotivated behaviour. Neuropsychologia. 2018;118:54–67. 28689673 10.1016/j.neuropsychologia.2017.07.003 PMC6200857

[6] Huang C, Ravdin LD, Nirenberg MJ, et al Neuroimaging markers of motor and nonmotor features of Parkinson’s disease: An 18f fluorodeoxyglucose positron emission computed tomography study. Dement Geriatr Cogn Disord. 2013;35(3–4):183–196. 23445555 10.1159/000345987

[7] Carriere N, Besson P, Dujardin K, et al Apathy in Parkinson’s disease is associated with nucleus accumbens atrophy: A magnetic resonance imaging shape analysis. Mov Disord. 2014;29(7):897–903. 24817690 10.1002/mds.25904

[8] Wen MC, Chan LL, Tan LCS, Tan EK. Depression, anxiety, and apathy in Parkinson’s disease: Insights from neuroimaging studies. Eur J Neurol. 2016;23(6):1001–1019. 27141858 10.1111/ene.13002 PMC5084819

[9] Theleritis C, Politis A, Siarkos K, Lyketsos CG. A review of neuroimaging findings of apathy in Alzheimer’s disease. Int Psychogeriatr. 2014;26(2):195–207. 24135083 10.1017/S1041610213001725 PMC4086515

[10] Muhammed K, Ben YM, Drew D, Manohar S, Husain M. Reward sensitivity and action in Parkinson’s disease patients with and without apathy. Brain Commun. 2021;3(2):fcab022. 33855297 10.1093/braincomms/fcab022 PMC8024004

[11] Lawrence AD, Goerendt IK, Brooks DJ. Apathy blunts neural response to money in Parkinson’s disease. Soc Neurosci. 2011;6(5–6):653–662. 21400357 10.1080/17470919.2011.556821

[12] Robert GH, Le Jeune F, Lozachmeur C, et al Preoperative factors of apathy in subthalamic stimulated Parkinson disease: A PET study. Neurology. 2014;83(18):1620–1626. 25253750 10.1212/WNL.0000000000000941

[13] Remy P, Doder M, Lees A, Turjanski N, Brooks D. Depression in Parkinson’s disease: Loss of dopamine and noradrenaline innervation in the limbic system. Brain. 2005;128(Pt 6):1314–1322. 15716302 10.1093/brain/awh445

[14] Thobois S, Ardouin C, Lhommée E, et al Non-motor dopamine withdrawal syndrome after surgery for Parkinson’s disease: Predictors and underlying mesolimbic denervation. Brain. 2010;133(Pt 4):1111–1127. 20237128 10.1093/brain/awq032

[15] Pagonabarraga J, Kulisevsky J, Strafella AP, Krack P. Apathy in Parkinson’s disease: Clinical features, neural substrates, diagnosis, and treatment. Lancet Neurol. 2015;14(5):518–531. 25895932 10.1016/S1474-4422(15)00019-8

[16] Schmidt L, Lebreton M, Cléry-Melin ML, Daunizeau J, Pessiglione M. Neural mechanisms underlying motivation of mental versus physical effort. PLoS Biol. 2012;10(2):e1001266. 22363208 10.1371/journal.pbio.1001266 PMC3283550

[17] Chong TTJ, Apps M, Giehl K, Sillence A, Grima LL, Husain M. Neurocomputational mechanisms underlying subjective valuation of effort costs. PLoS Biol. 2017;15(2):e1001266. 10.1371/journal.pbio.1002598 PMC5325181 28234892

[18] Bonnelle V, Manohar S, Behrens T, Husain M. Individual differences in premotor brain systems underlie behavioral apathy. Cereb Cortex. 2016;26(2):807–819. 26564255 10.1093/cercor/bhv247 PMC4712805

[19] Le Heron C, Plant O, Manohar S, et al Distinct effects of apathy and dopamine on effort-based decision-making in Parkinson’s disease. Brain. 2018;141(5):1455–1469. 29672668 10.1093/brain/awy110 PMC5917786

[20] Muhammed K, Manohar S, Ben Yehuda M, et al Reward sensitivity deficits modulated by dopamine are associated with apathy in Parkinson’s disease. Brain. 2016;139(Pt 10):2706–2721. 27452600 10.1093/brain/aww188 PMC5035817

[21] Frömer R, Lin H, Dean Wolf CK, Inzlicht M, Shenhav A. Expectations of reward and efficacy guide cognitive control allocation. Nat Commun. 2021;12(1):1030. 33589626 10.1038/s41467-021-21315-z PMC7884731

[22] Wilhelm RA, Miller MW, Gable PA. Neural and attentional correlates of intrinsic motivation resulting from social performance expectancy. Neuroscience. 2019;416:137–146. 31369789 10.1016/j.neuroscience.2019.07.039

[23] Gable PA, Threadgill AH, Adams DL. Neural activity underlying motor-action preparation and cognitive narrowing in approach-motivated goal states. Cogn Affect Behav Neurosci. 2016;16(1):145–152. 26453581 10.3758/s13415-015-0381-4

[24] Meyniel F, Pessiglione M. Better get back to work: A role for motor beta desynchronization in incentive motivation. J Neurosci. 2014;34(1):1–9. 24381263 10.1523/JNEUROSCI.1711-13.2014 PMC6608166

[25] Brown P . Bad oscillations in Parkinson’s disease. J Neural Transm Suppl. 2006;70:27–30. 10.1007/978-3-211-45295-0_6 17017505

[26] Brown P . Oscillatory nature of human basal ganglia activity: Relationship to the pathophysiology of Parkinson’s disease. Mov Disord. 2003;18(4):357–363. 12671940 10.1002/mds.10358

[27] Brown P . Abnormal oscillatory synchronisation in the motor system leads to impaired movement. Curr Opin Neurobiol. 2007;17(6):656–664. 18221864 10.1016/j.conb.2007.12.001

[28] Engel AK, Moll CKE, Fried I, Ojemann GA. Invasive recordings from the human brain: Clinical insights and beyond. Nat Rev Neurosci. 2005;6(1):35–47. 15611725 10.1038/nrn1585

[29] Heinrichs-Graham E, Wilson TW, Santamaria PM, et al Neuromagnetic evidence of abnormal movement-related beta desynchronization in Parkinson’s disease. Cereb Cortex. 2014;24(10):2669–2678. 23645717 10.1093/cercor/bht121 PMC4153806

[30] Lofredi R, Tan H, Neumann WJ, et al Beta bursts during continuous movements accompany the velocity decrement in Parkinson’s disease patients. Neurobiol Dis. 2019;127:462–471. 30898668 10.1016/j.nbd.2019.03.013 PMC6520224

[31] Prokic EJ, Stanford IM, Woodhall GL, Williams AC, Hall SD. Bradykinesia is driven by cumulative beta power during continuous movement and alleviated by GABAergic modulation in Parkinson’s disease. Front Neurol. 2019;10:1298. 31920922 10.3389/fneur.2019.01298 PMC6933612

[32] Berardelli A, Rothwell JC, Thompson PD, Hallett M. Pathophysiology of bradykinesia in Parkinson’s disease. Brain. 2001;124(Pt 11):2131–2146. 11673316 10.1093/brain/124.11.2131

[33] Eisinger RS, Cagle JN, Opri E, et al Parkinsonian beta dynamics during rest and movement in the dorsal pallidum and subthalamic nucleus. J Neurosci. 2020;40(14):2859–2867. 32107277 10.1523/JNEUROSCI.2113-19.2020 PMC7117906

[34] Martínez-Horta S, Riba J, de Bobadilla RF, et al Apathy in Parkinson’s disease: Neurophysiological evidence of impaired incentive processing. J Neurosci. 2014;34(17):5918–5926. 24760851 10.1523/JNEUROSCI.0251-14.2014 PMC6608288

[35] Zhu M, HajiHosseini A, Baumeister TR, Garg S, Appel-Cresswell S, McKeown MJ. Altered EEG alpha and theta oscillations characterize apathy in Parkinson’s disease during incentivized movement. Neuroimage Clin. 2019;23:101922. 31284232 10.1016/j.nicl.2019.101922 PMC6614604

[36] Lobaugh NJ, West R, McIntosh AR. Spatiotemporal analysis of experimental differences in event-related potential data with partial least squares. Psychophysiology. 2001;38(3):517–530. 11352141 10.1017/s0048577201991681

[37] Kilavik BE, Zaepffel M, Brovelli A, MacKay WA, Riehle A. The ups and downs of beta oscillations in sensorimotor cortex. Exp Neurol. 2013;245:15–26. 23022918 10.1016/j.expneurol.2012.09.014

[38] Jenkinson N, Brown P. New insights into the relationship between dopamine, beta oscillations and motor function. Trends Neurosci. 2011;34(12):611–618. 10.1016/j.tins.2011.09.003 22018805

[39] Barone J, Rossiter HE. Understanding the role of sensorimotor beta oscillations. Front Syst Neurosci. 2021;15:655886. 34135739 10.3389/fnsys.2021.655886 PMC8200463

[40] Gómez-Herrero G, De Clercq W, Anwar H, et al *Automatic removal of ocular artifacts in the EEG without an EOG reference channel*. In: *Proceedings of the 7th Nordic Signal Processing Symposium, NORSIG*. IEEE;2006:130–133.

[41] Makeig S, Jung TP, Bell AJ, Sejnowski TJ. Independent component analysis of electroencephalographic data. Adv Neural Inf Process Syst. 1996;8(8):145–151.

[42] Correa N, Adali T, Li YO, Calhoun V. Canonical correlation analysis for data fusion and group inferences. IEEE Signal Process Mag. 2010;27(4):39–50. 20706554 10.1109/MSP.2010.936725 PMC2919827

[43] Li Y-O, Adali T, Wang W, Calhoun VD. Joint blind source separation by multiset canonical correlation analysis. IEEE Trans Signal Process. 2009;57(10):3918–3929. 20221319 10.1109/TSP.2009.2021636 PMC2835373

[44] McFarland DJ, Miner LA, Vaughan TM, Wolpaw JR. Mu and beta rhythm topographies during motor imagery and actual movements. Brain Topogr. 2000;12(3):177–186. 10791681 10.1023/a:1023437823106

[45] Zaepffel M, Trachel R, Kilavik BE, Brochier T. Modulations of EEG beta power during planning and execution of grasping movements. PLoS One. 2013;8(3):e60060. 23555884 10.1371/journal.pone.0060060 PMC3605373

[46] Nelson AB, Moisello C, Lin J, et al Beta oscillatory changes and retention of motor skills during practice in healthy subjects and in patients with Parkinson’s disease. Front Hum Neurosci. 2017;11:104. 28326029 10.3389/fnhum.2017.00104 PMC5339296

[47] Pirondini E, Coscia M, Minguillon J, Millán JDR, Van De Ville D, Micera S. EEG topographies provide subject-specific correlates of motor control. Sci Rep. 2017;7(1):13229. 29038516 10.1038/s41598-017-13482-1 PMC5643537

[48] Choi JW, Malekmohammadi M, Sparks H, et al Altered pallidocortical low-beta oscillations during self-initiated movements in Parkinson disease. Front Syst Neurosci. 2020;14:54. 32792918 10.3389/fnsys.2020.00054 PMC7390921

[49] van Wijk BCM, Neumann WJ, Schneider GH, Sander TH, Litvak V, Kühn AA. Low-beta cortico-pallidal coherence decreases during movement and correlates with overall reaction time. Neuroimage. 2017;159:1–8. 28712991 10.1016/j.neuroimage.2017.07.024 PMC5678295

[50] Savoie FA, Hamel R, Lacroix A, Thénault F, Whittingstall K, Bernier PM. Luring the motor system: Impact of performance-contingent incentives on pre-movement beta-band activity and motor performance. J Neurosci. 2019;39(15):2903–2914. 30737309 10.1523/JNEUROSCI.1887-18.2019 PMC6462448

[51] Tzagarakis C, Ince NF, Leuthold AC, Pellizzer G. Beta-band activity during motor planning reflects response uncertainty. J Neurosci. 2010;30(34):11270–11277. 20739547 10.1523/JNEUROSCI.6026-09.2010 PMC6633326

[52] Kilner JM, Bott L, Posada A. Modulations in the degree of synchronization during ongoing oscillatory activity in the human brain. Eur J Neurosci. 2005;21(9):2547–2554. 15932612 10.1111/j.1460-9568.2005.04069.x

[53] Kaiser J, Birbaumer N, Lutzenberger W. Event-related beta desynchronization indicates timing of response selection in a delayed-response paradigm in humans. Neurosci Lett. 2001;312(3):149–152. 11602332 10.1016/s0304-3940(01)02217-0

[54] Baker SN . Oscillatory interactions between sensorimotor cortex and the periphery. Curr Opin Neurobiol. 2007;17(6):649–655. 18339546 10.1016/j.conb.2008.01.007 PMC2428102

[55] Pfurtscheller G, Lopes da Silva FH. Event-related EEG/MEG synchronization and desynchronization: Basic principles. Clin Neurophysiol. 1999;110(11):1842–1857. 10576479 10.1016/s1388-2457(99)00141-8

[56] Kühn AA, Williams D, Kupsch A, et al Event-related beta desynchronization in human subthalamic nucleus correlates with motor performance. Brain. 2004;127(4):735–746. 14960502 10.1093/brain/awh106

[57] Gilbertson T, Lalo E, Doyle L, Di Lazzaro V, Cioni B, Brown P. Existing motor state is favored at the expense of new movement during 13–35 Hz oscillatory synchrony in the human corticospinal system. J Neurosci. 2005;25(34):7771–7779. 16120778 10.1523/JNEUROSCI.1762-05.2005 PMC6725263

[58] Androulidakis AG, Doyle LMF, Gilbertson TP, Brown P. Corrective movements in response to displacements in visual feedback are more effective during periods of 13–35 Hz oscillatory synchrony in the human corticospinal system. Eur J Neurosci. 2006;24(11):3299–3304. 17156390 10.1111/j.1460-9568.2006.05201.x

[59] Androulidakis AG, Doyle LMF, Yarrow K, Litvak V, Gilbertson TP, Brown P. Anticipatory changes in beta synchrony in the human corticospinal system and associated improvements in task performance. Eur J Neurosci. 2007;25(12):3758–3765. 17610595 10.1111/j.1460-9568.2007.05620.x

[60] Pogosyan A, Gaynor LD, Eusebio A, Brown P. Boosting cortical activity at beta-band frequencies slows movement in humans. Curr Biol. 2009;19(19):1637–1641. 19800236 10.1016/j.cub.2009.07.074 PMC2791174

[61] Engel AK, Fries P. Beta-band oscillations—Signalling the status quo? Curr Opin Neurobiol. 2010;20(2):156–165. 20359884 10.1016/j.conb.2010.02.015

[62] Brown P, Marsden CD. What do the basal ganglia do? Lancet. 1998;351(9118):1801–1804. 9635969 10.1016/s0140-6736(97)11225-9

[63] Wang HC, Lees AJ, Brown P. Impairment of EEG desynchronisation before and during movement and its relation to bradykinesia in Parkinson’s disease. J Neurol Neurosurg Psychiatry. 1999;66(4):442–446. 10201414 10.1136/jnnp.66.4.442 PMC1736289

[64] Brown P, Marsden D. Bradykinesia and impairment of EEG desynchronization in Parkinson’s disease. Mov Disord. 1999;14(3):423–429. 10348464 10.1002/1531-8257(199905)14:3<423::aid-mds1006>3.0.co;2-v

[65] Stegemöller EL, Allen DP, Simuni T, MacKinnon CD. Motor cortical oscillations are abnormally suppressed during repetitive movement in patients with Parkinson’s disease. Clin Neurophysiol. 2016;127(1):664–674. 26089232 10.1016/j.clinph.2015.05.014 PMC6106791

[66] Shirahige L, Berenguer-Rocha M, Mendonça S, Rocha S, Rodrigues MC, Monte-Silva K. Quantitative electroencephalography characteristics for Parkinson’s disease: A systematic review. J Parkinsons Dis. 2020;10(2):455–470. 32065804 10.3233/JPD-191840 PMC7242841

[67] Zimmermann R, Gschwandtner U, Hatz F, et al Correlation of EEG slowing with cognitive domains in nondemented patients with Parkinson’s disease. Dement Geriatr Cogn Disord. 2015;39(3–4):207–214. 25591733 10.1159/000370110

[68] Geraedts VJ, Boon LI, Marinus J, et al Clinical correlates of quantitative EEG in Parkinson disease: A systematic review. Neurology. 2018;91(19):871–883. 30291182 10.1212/WNL.0000000000006473

[69] Vecchio F, Babiloni C, Lizio R, et al Resting state cortical EEG rhythms in Alzheimer’s disease: Toward EEG markers for clinical applications: A review. Suppl Clin Neurophysiol. 2013;62:223–236. 24053043 10.1016/b978-0-7020-5307-8.00015-6

[70] Babiloni C, De Pandis MF, Vecchio F, et al Cortical sources of resting state electroencephalographic rhythms in Parkinson’s disease related dementia and Alzheimer’s disease. Clin Neurophysiol. 2011;122(12):2355–2364. 21924950 10.1016/j.clinph.2011.03.029

[71] Fonseca LC, Tedrus GMAS, Carvas PN, Machado ECFA. Comparison of quantitative EEG between patients with Alzheimer’s disease and those with Parkinson’s disease dementia. Clin Neurophysiol. 2013;124(10):1970–1974. 23746496 10.1016/j.clinph.2013.05.001

[72] Padmanabhapillai A, Porjesz B, Ranganathan M, et al Suppression of early evoked gamma band response in male alcoholics during a visual oddball task. Int J Psychophysiol. 2006;60(1):15–26. 16019097 10.1016/j.ijpsycho.2005.03.026

[73] Farzan F, Barr MS, Wong W, Chen R, Fitzgerald PB, Daskalakis ZJ. Suppression of gamma-oscillations in the dorsolateral prefrontal cortex following long interval cortical inhibition: A TMS-EEG study. Neuropsychopharmacology. 2009;34(6):1543–1551. 19037204 10.1038/npp.2008.211

[74] Wang Q, Meng L, Pang J, Zhu X, Ming D. Characterization of EEG data revealing relationships with cognitive and motor symptoms in Parkinson’s disease: A systematic review. Front Aging Neurosci. 2020;12:587396. 33240076 10.3389/fnagi.2020.587396 PMC7683572

[75] Bočková M, Rektor I. Impairment of brain functions in Parkinson’s disease reflected by alterations in neural connectivity in EEG studies: A viewpoint. Clin Neurophysiol. 2019;130(2):239–247. 30580247 10.1016/j.clinph.2018.11.013

[76] Olde Dubbelink KTE, Stoffers D, Deijen JB, Twisk JWR, Stam CJ, Berendse HW. Cognitive decline in Parkinson’s disease is associated with slowing of resting-state brain activity: A longitudinal study. Neurobiol Aging. 2013;34(2):408–418. 22495052 10.1016/j.neurobiolaging.2012.02.029

[77] Xie J, Peng M, Lu J, et al Enhancement of event-related desynchronization in motor imagery based on transcranial electrical stimulation. Front Hum Neurosci. 2021;15:635351. 33815080 10.3389/fnhum.2021.635351 PMC8012503

