# Supplementary material for: Apathy scores in Parkinson’s disease relate to EEG components in an incentivized motor task
Source: Brain Commun. 2024 Feb 9;6(1):fcae025. doi: 10.1093/braincomms/fcae025 (PMC10873141; doi:10.1093/braincomms/fcae025)
Supplement: fcae025_Supplementary_Data [file fcae025_supplementary_data.docx]

**Supplementary material**


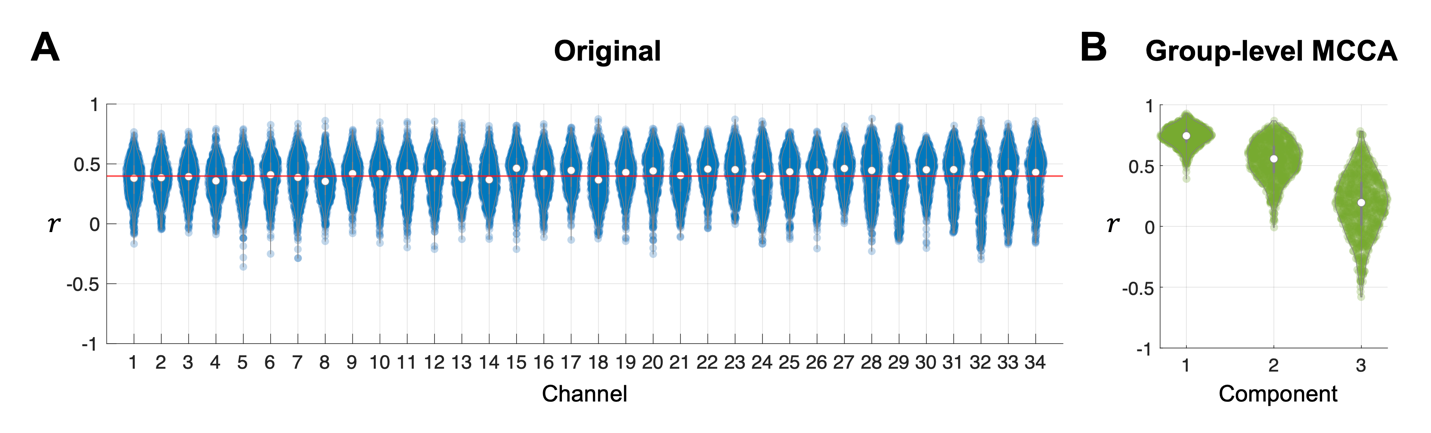


**Supplementary Fig. 1.** **Between-subject correlations of the ERSPs.** Correlations between the ERSPs of every pair of two participants were computed for each channel or component. **(A)** Distributions of the between-subject correlations of the original ERSPs ($Y_{s}$) across 34 channels. The grand mean of the correlations of all channels is denoted as a red line. **(B)** Distributions of the between-subject correlations of the group-level MCCA component ($P_{s}$). ERSP: event-related spectral perturbation; MCCA: multi-set canonical correlation analysis.


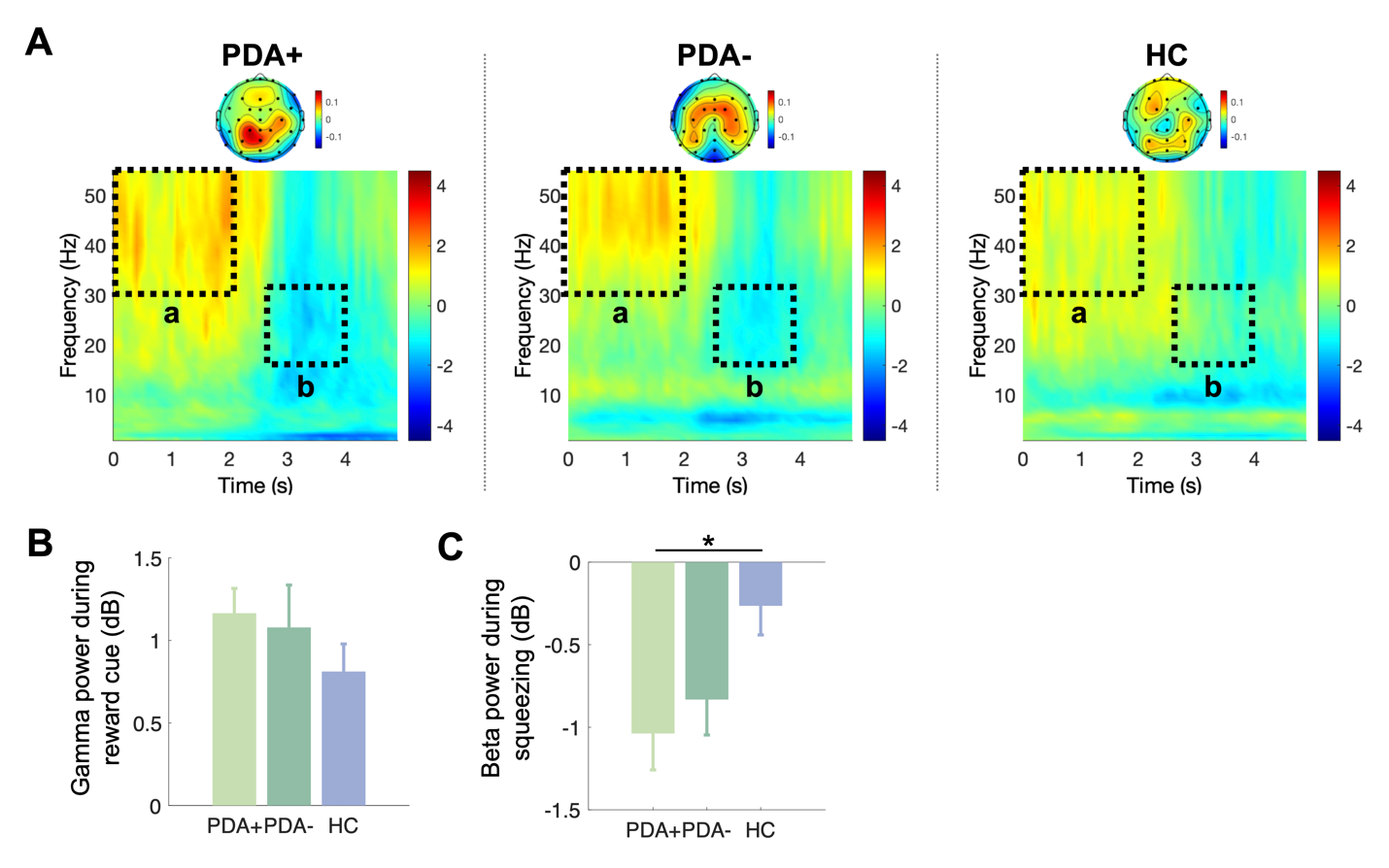


**Supplementary Fig. 2.** **Third group-level MCCA component.** **(A)** The ERSP and its weights across EEG channels are demonstrated as a scalogram (bottom) and scalp map (top) for each group. The gamma (30–55 Hz) frequency band during the reward cue (**a**: 0–5 s) and beta (12–30 Hz) frequency band during squeezing (**b**: 2.5–4 s) are denoted as dotted boxes. **(B)** Group comparison of the gamma power during the reward cue (statistics: one-way ANOVA). **(C)** Group comparison of the beta power during squeezing (statistics: one-way ANOVA, Tukey’s honestly significant difference test). *$P$ < 0.05. ERSP: event-related spectral perturbation; HC: healthy controls (N = 12); MCCA: multi-set canonical correlation analysis; PDA+: Parkinson’s disease patients with apathy (N = 13); PDA-: Parkinson’s disease patients without apathy (N = 13).


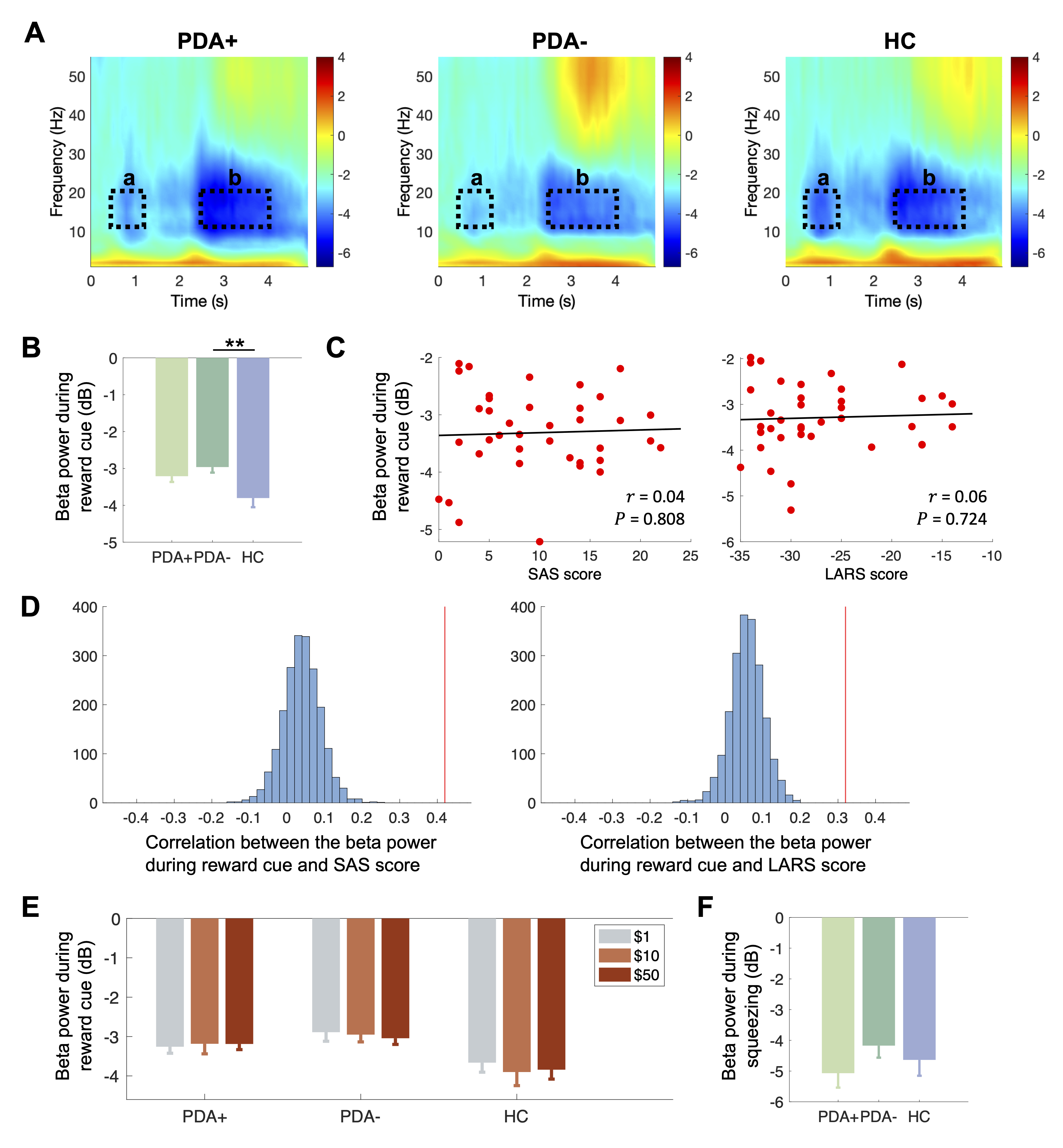


**Supplementary Fig. 3. ERSP results from the conventional analysis**. **(A)** The group mean ERSPs over the central-parietal channels (C3, CZ, C4, CP5, CP1, CPZ, CP2, CP6, P3, PZ, and P4). The low-beta (12–20 Hz) frequency band during a reward cue (**a**: 0.3–1 s) and squeezing (**b**: 2.5–4 s) is denoted as dotted boxes. **(B)** Group comparison of the beta power during reward cue (statistics: one-way ANOVA, Tukey’s honestly significant difference test). **(C)** Correlations between the beta power during reward cue and clinical apathy scores (SAS and LARS). **(D)** Distributions of the correlations between the beta power during reward cue and clinical apathy scores when the beta power was computed for every possible subset of the central-parietal channels. The red lines indicate the correlation values obtained from the first MCCA component (Fig. 5C) for comparison. **(E)** The beta power during reward cue is presented per reward level for each group (statistics: Friedman test). **(F)** Group comparison of the beta power during squeezing (one-way ANOVA). **$P$ < 0.01. ERSP: event-related spectral perturbation; HC: healthy controls (N = 12); LARS: Lille apathy rating scale; MCCA: multi-set canonical correlation analysis; PDA+: Parkinson’s disease patients with apathy (N = 13); PDA-: Parkinson’s disease patients without apathy (N = 13); SAS: Starkstein apathy scale.


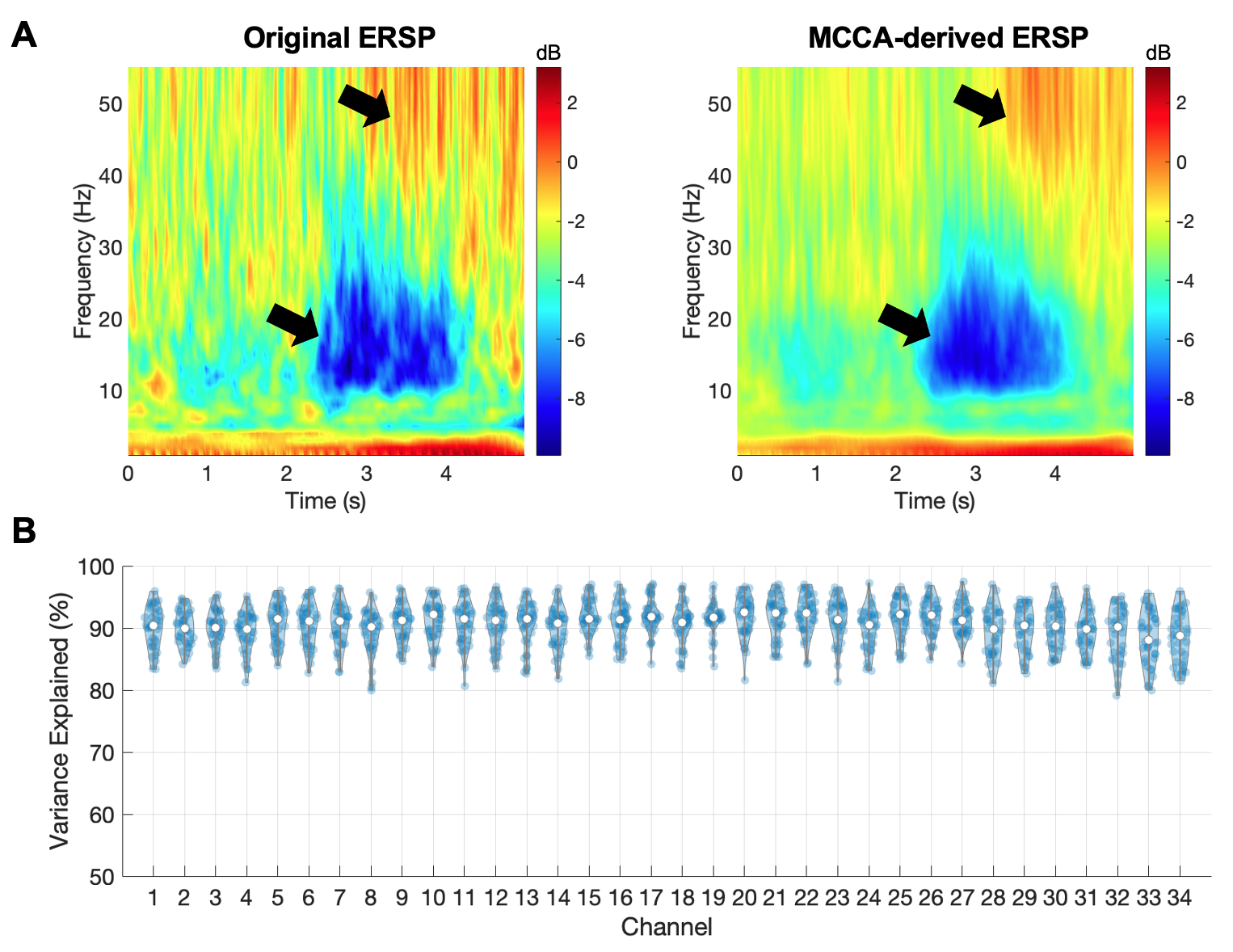


**Supplementary Fig. 4. Qualitative and quantitative comparison of the original ERSP and ERSP derived from MCCA (both sampled at 50 Hz)**. **(A)** Left: the trial-mean ERSP ($Y_{s}$ in Fig. 3) of channel FP1 from the same representative participant presented in Fig. 4 is shown in left. Right: The MCCA-derived ERSP ($X_{s}$ in Fig. 3) preserves task-relevant spectral changes in the original ERSP as denoted by arrows. **(B)** The distribution of the variance of the original ERSP explained by the MCCA-derived ERSP computed for each participant per channel. ERSP: event-related spectral perturbation; MCCA: multi-set canonical correlation analysis.


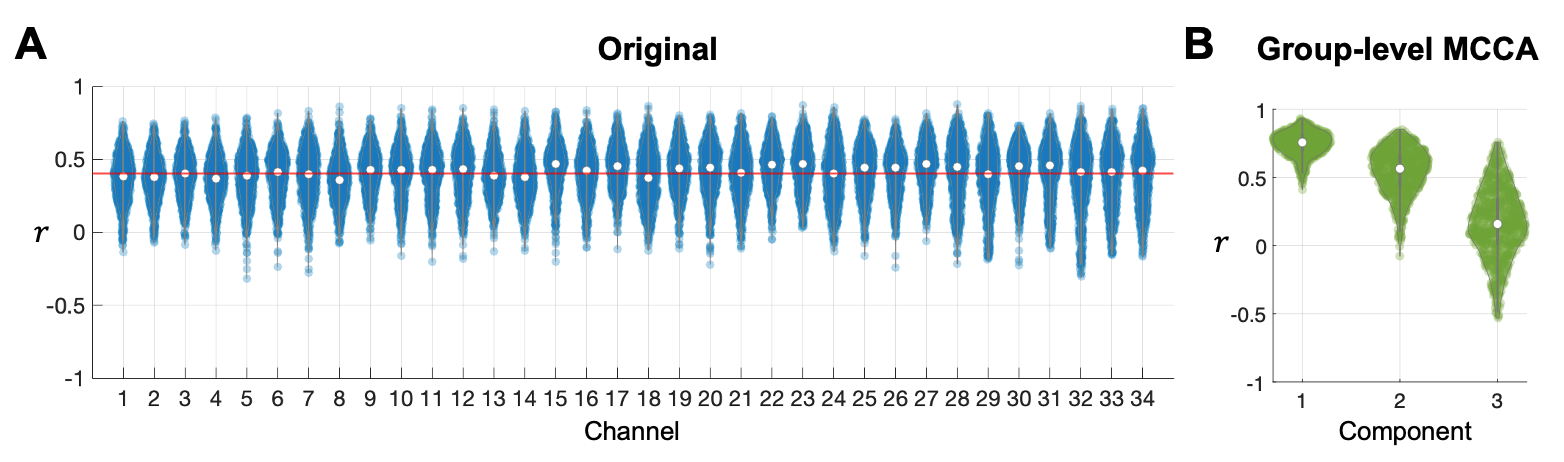


**Supplementary Fig. 5. Between-subject correlations of the ERSPs sampled at 50 Hz**. Correlations between the ERSPs of every pair of two participants were computed for each channel or component. **(A)** Distributions of the between-subject correlations of the original ERSPs ($Y_{s}$) across 34 channels. The grand mean of the correlations of all channels is denoted as a red line. **(B)** Distributions of the between-subject correlations of the group-level MCCA component ($P_{s}$). ERSP: event-related spectral perturbation; MCCA: multi-set canonical correlation analysis.


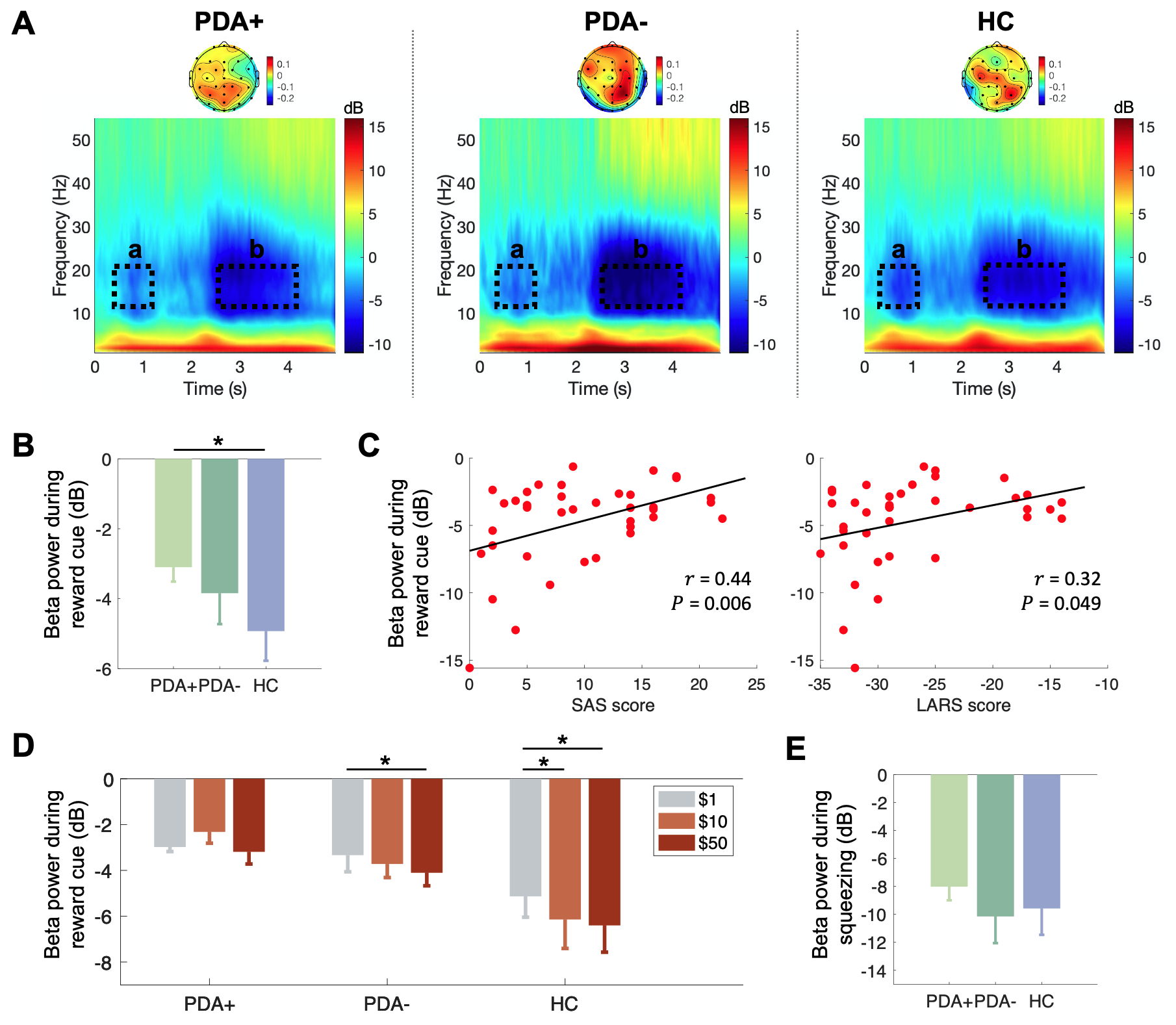


**Supplementary Fig. 6. First group-level MCCA component obtained from the ERSPs sampled at 50 Hz.** **(A)** The ERSP and its weights across EEG channels are demonstrated as a scalogram (bottom) and scalp map (top) for each group. The low-beta (12–20 Hz) frequency band during a reward cue (**a**: 0.3–1 s) and squeezing (**b**: 2.5–4 s) is denoted as dotted boxes. **(B)** Group comparison of the beta power during a reward cue (statistics: one-way ANOVA, Tukey’s honestly significant difference test). **(C)** Correlations between the beta power during a reward cue and clinical apathy scores (SAS and LARS) of all the participants. **(D)** The beta power during the reward cue is presented per reward level for each group (statistics: Friedman test, Wilcoxon signed-rank test). **(E)** Group comparison of the beta power during squeezing (statistics: one-way ANOVA). *$P$ < 0.05. ERSP: event-related spectral perturbation; HC: healthy controls (N = 12); LARS: Lille apathy rating scale; MCCA: multi-set canonical correlation analysis; PDA+: Parkinson’s disease patients with apathy (N = 13); PDA-: Parkinson’s disease patients without apathy (N = 13); SAS: Starkstein apathy scale.


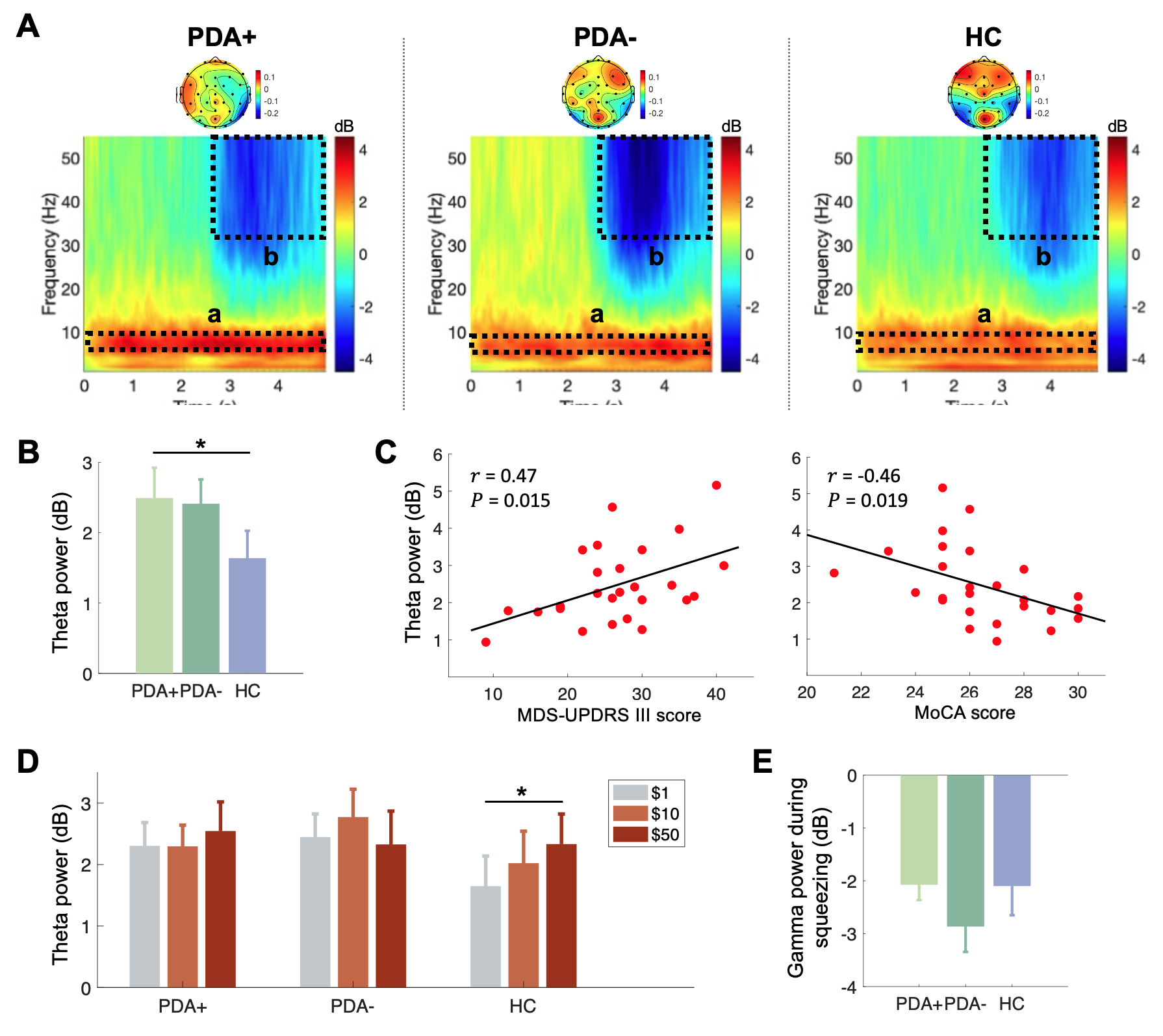


**Supplementary Fig. 7. Second group-level MCCA component obtained from the ERSPs sampled at 50 Hz.** **(A)** The ERSP and its weights across EEG channels are demonstrated as a scalogram (bottom) and scalp map (top) for each group. The theta (4–8 Hz) frequency band during the reward cue and squeezing (**a**: 0–5 s) and gamma (30–55 Hz) frequency band during squeezing (**b**: 2.5–5 s) are denoted as dotted boxes. **(B)** Group comparison of the theta power during the reward cue and squeezing (statistics: one-way ANOVA, Tukey’s honestly significant difference test). **(C)** Correlation between the theta power and MDS-UPDRS Part III scores (left) and between the theta power and MoCA scores (right) of the PD participants. **(D)** The theta power is presented per reward level for each group (statistics: Friedman test, Wilcoxon signed-rank test). **(E)** Group comparison of the gamma power during squeezing (statistics: one-way ANOVA). *$P$ < 0.05. ERSP: event-related spectral perturbation; HC: healthy controls (N = 12); LARS: Lille apathy rating scale; MCCA: multi-set canonical correlation analysis; MDS-UPDRS: movement disorder society-unified Parkinson's disease rating scale; MoCA: Montreal cognitive assessment; PD: Parkinson’s disease; PDA+: Parkinson’s disease patients with apathy (N = 13); PDA-: Parkinson’s disease patients without apathy (N = 13); SAS: Starkstein apathy scale.


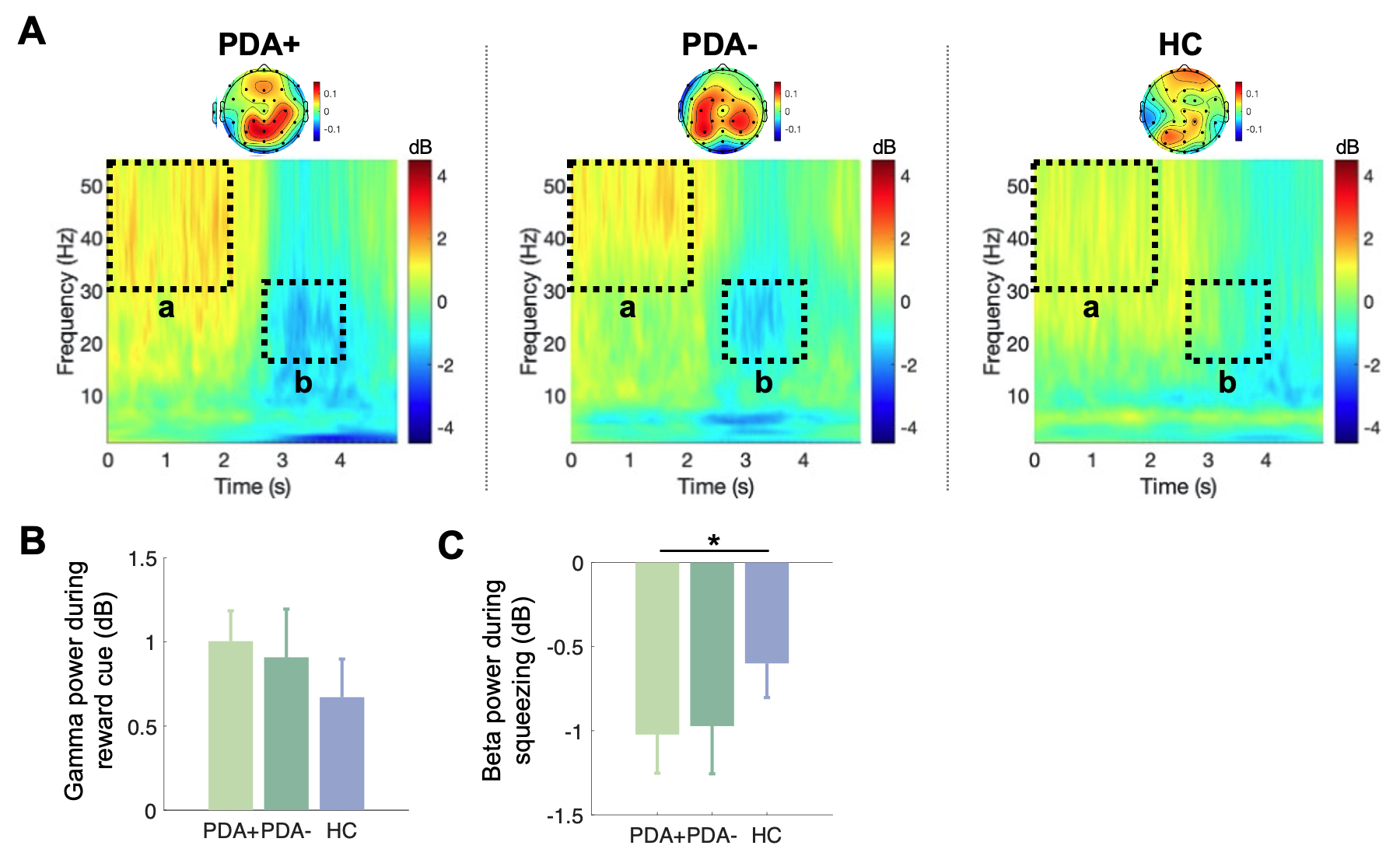


**Supplementary Fig. 8. Third group-level MCCA component obtained from the ERSPs sampled at 50 Hz.** **(A)** The ERSP and its weights across EEG channels are demonstrated as a scalogram (bottom) and scalp map (top) for each group. The gamma (30–55 Hz) frequency band during the reward cue (**a**: 0–5 s) and beta (12–30 Hz) frequency band during squeezing (**b**: 2.5–4 s) are denoted as dotted boxes. **(B)** Group comparison of the gamma power during the reward cue (statistics: one-way ANOVA). **(C)** Group comparison of the beta power during squeezing (statistics: one-way ANOVA, Tukey’s honestly significant difference test). *$P$ < 0.05. ERSP: event-related spectral perturbation; HC: healthy controls (N = 12); MCCA: multi-set canonical correlation analysis; PDA+: Parkinson’s disease patients with apathy (N = 13); PDA-: Parkinson’s disease patients without apathy (N = 13).
